# The Influence of Wild Grapevine Endophytes on the Growth of the Model Plant *Arabidopsis thaliana* (L.) Heynh

**DOI:** 10.3390/life16040566

**Published:** 2026-03-31

**Authors:** Olga A. Aleynova, Alexey A. Ananev, Nikolay N. Nityagovsky, Konstantin V. Kiselev

**Affiliations:** Laboratory of Biotechnology, Federal Scientific Center of the East Asia Terrestrial Biodiversity, Far Eastern Branch of the Russian Academy of Sciences, 690022 Vladivostok, Russia; ananev.all@yandex.ru (A.A.A.); niknit1996@gmail.com (N.N.N.); kiselev@biosoil.ru (K.V.K.)

**Keywords:** fungi, bacteria, microbiome, *Vitis amurensis*, plant growth promotion, plant biostimulants

## Abstract

We have evaluated the growth characteristics of the model plant *Arabidopsis thaliana* after inoculation of *A. thaliana* seeds with the most common endophytes of wild grapevine *Vitis amurensis* Rupr., namely bacteria *Rhizobium* (syn. *Agrobacterium*) sp., *Bacillus velezensis*, *Curtobacterium* sp, *Erwinia* sp., *Gordonia aichiensis*, *Pantoea* sp., *Pseudomonas* sp., *Sphingomonas* sp., *Xanthomonas* sp., and the fungi *Biscogniauxia* sp., *Cladosporium* sp., *Didymella* sp., *Exobasidium* sp., *Penicillium* sp., *Pestalotiopsis* sp, and *Xylaria* sp. A positive effect on plant growth was observed in *A. thaliana* following seed inoculation with endophytic fungi (*Xylaria* sp., *Didymella* sp., and *Exobasidium* sp.) and bacteria (*Gordonia aichiensis* and *Sphingomonas* sp.). The inoculation with the fungi *Xylaria* sp., *Didymella* sp., *Exobasidium* sp., and *Penicillium* sp. significantly increased seed production in *A. thaliana* by 2.5–5-fold. The analysis of the phytohormone-regulating gene transcription in *A. thaliana* plants following inoculation with the grapevine endophytic microorganisms suggests that plant growth was enhanced through transcriptional changes in individual genes of hormone biosynthetic pathways. Consequently, endophytic bacteria and fungi from *V. amurensis* may serve as potential natural growth stimulators for agricultural plants.

## 1. Introduction

Plants are closely associated with microorganisms, residing in all tissues [1]. A special group of microorganisms associated with plants is endophytes—microorganisms that inhabit the internal plant tissues [2]. Endophytes inhabit all parts of plant organs: roots, leaves, stems, flowers, and seeds. Endophytes can enter a plant through its root system, aboveground parts, cracks, seeds, or vegetative plant propagation [2]. Some endophytes may act as latent pathogens, while others may play an essential role in maintaining plant health. Endophytes can protect or prepare the plant against abiotic and biotic stresses and help in enhancing growth and yields [3,4].

The remarkable ability of endophytes to promote plant growth stems from their diverse mechanisms of action, encompassing both direct and indirect pathways [5,6,7,8]. Direct growth-promoting methods consist of the production of compounds beneficial to plants, such as phytohormones, 1-aminocyclopropane-1-carboxylate (ACC) deaminase, iron sequestration and phosphate solubilization [7,8]. Plant hormones such as auxins, cytokinins (CKs), gibberellins (GAs), abscisic acid (ABA) and ethylene (ET) have a major impact on plant growth [9]. Multiple studies have corroborated that endophytes may produce plant hormones, including auxin, CKs, GAs, ABA and ET [10,11,12,13,14,15,16]. Endophytes can stimulate plant growth not only by directly producing their own phytohormones but also by activating a cascade of differential expression of host plant genes, which can additionally activate plant phytohormone biosynthesis genes [17,18].

It is important to note that some endophytes demonstrate broad host compatibility, while others exhibit high specificity, influenced by factors such as plant physiology, root exudates, immune responses, and environmental conditions [17,19]. The interaction of endophytes and plants is influenced by the condition of the plant, species, geographical location, climatic conditions, and even the season of sampling [20,21] [Rodriguez-Blanco et al., 2015; Ding and Melcher, 2016]. It was found that in some endophytes, the transition to a pathogenic lifestyle depends not only on local abiotic stress factors but also on the genotype of the host plant [22]. For example, fluorescent pseudomonads are commonly regarded as beneficial endophytic bacteria that promote plant growth. Studies have shown that, under certain conditions, they can have harmful effects on leatherleaf ferns [23]. This highlights a key ecological nuance: even microbes typically considered mutualistic may act as pathogens or stressors, depending on the host species and environmental context [24].

Grapes are among the most economically significant crops and widely cultivated fruit plants worldwide. Grapevines harbor a diverse community of microorganisms, including bacterial and fungal endophytes, which contribute to the formation of the so-called “terroir” and influence the qualities and characteristics of grapes and wine [25]. Some grapevine endophytes, in addition to their anti-pathogenic properties, are able to influence plant growth [26,27,28].

*V. amurensis* is a grapevine species native to Asia and is recognized as a rich source of secondary metabolites with potent antioxidant, anti-inflammatory, antibacterial, and cardioprotective properties [29,30]. Moreover, its use as a rootstock provides a valuable opportunity to develop grape cultivars with enhanced resistance to a broad spectrum of biotic and abiotic stresses. Therefore, investigating the endophytic microbiota isolated from the wild grapevine *V. amurensis* is of significant interest, as these microbes may contribute to the plants’ resilience and offer novel biocontrol or growth-promoting agents.

It has previously been shown that the main representatives of endophytes from aerial tissues of *V. amurensis* (leaf, stem, seed and berry) are bacteria of the classes *Gammaproteobacteria*, *Alphaproteobacteria*, *Actinobacteria*, *Bacilli*, and *Bacteroidia* [31], and fungi *Dothideomycetes* and *Tremellomycetes* [32]. The main endophytes of *V. amurensis* grapevine include representatives of the bacterial genera *Rhizobium* (syn. *Agrobacterium*) sp., *Bacillus* sp., *Curtobacterium* sp., *Erwinia* sp., *Gordonia* sp., *Pantoea* sp., *Pseudomonas* sp., *Sphingomonas* sp., and *Xanthomonas* sp., and fungal genera *Biscogniauxia* sp., *Cladosporium* sp., *Didymella* sp., *Exobasidium* sp., *Penicillium* sp., *Pestalotiopsis* sp. and *Xylaria * sp. [32].

It was shown that some members of *Rhizobium*, *Bacillus*, *Curtobacterium*, *Gordonia*, *Pseudomonas* and *Sphingomonas* species are classified as endophytes, which establish themselves intracellularly in order to promote root growth through both direct and indirect mechanisms [33,34,35]. The genera *Erwinia*, *Pantoea* and *Xanthomonas* comprise bacteria associated with plants in various ecological roles, including as pathogens, saprophytes, epiphytes, and endophytes [36,37].

Plant-associated fungal isolates also exhibit the same range of functions as bacteria, with beneficial effects on plant growth. The representatives of the genera *Biscogniauxia*, *Cladosporium*, *Didymella*, *Exobasidium*, and *Penicillium* are endophytic, saprophytic, weakly parasitic and pathogenic species that are often found in different plants around the world [36,38]. Some *Biscogniauxia* fungi produce antibacterials that could be useful in biotechnology [39,40]. Some species of *Cladosporium* and *Penicillium* have the plant growth-promoting capacity and the potential to become a microbial fertilizer for sustainable crop production [41,42]. *Pestalotiopsis* is ordinarily isolated as endophytes in plants [43,44,45]. The genus *Xylaria* comprises various endophytic species associated with both vascular and non-vascular plants [46]. *Xylaria* sp. has broad antimicrobial activity [47].

Previously, we examined the effect of dominant endophytic bacteria and fungi isolated from wild grapevine *V. amurensis* on grape cell culture growth and the synthesis of resveratrol, a prominent biologically active compound with beneficial effects on human health [29]. Endophytic bacteria significantly increased total stilbene content by 2.2–5.3-fold, while endophytic fungi were more effective, enhancing stilbene accumulation by 2.6–16.3-fold [48]. These findings suggested that wild grapevine endophytic bacteria and fungi hold promise for enhancing the nutritional value and overall quality of agricultural products. However, it remains unknown whether *V. amurensis* endophytes function as growth-stimulating microorganisms and which pathways mediate their growth-stimulating effects.

Therefore, this study aimed to investigate the effects of dominant endophytic bacteria and fungi isolated from the wild grapevine *V. amurensis* on growth characteristics and phytohormone metabolism gene expression in the model plant *Arabidopsis thaliana* (L.) Heynh. *Arabidopsis* was selected due to its short life cycle (2–3 months) and its well-characterized functions of phytohormone-related genes.

## 2. Materials and Methods

### 2.1. Endophytic Bacteria and Fungi Isolation and Identification

Endophytic bacteria and fungi were isolated from superficially sterilized leaves and stems of wild grapevines, *V. amurensis*, growing on the unprotected regions of the Primorsky Territory of Russia. Approximately 1.5 g of leaf and stem tissue was washed with mild soap, followed by surface sterilization in 75% (*v*/*v*) ethanol for two minutes and then in 10% (*v*/*v*) hydrogen peroxide for 1 min. Samples were rinsed five times with sterile distilled water, and a sample of final rinse water was cultured on PDA (Neogene, UK) or R2A (Himedia, India) media to verify the efficacy of the sterilization protocol. The sterile leaves and stems were ground in a mortar until a homogeneous pulp was obtained. The resulting homogenate was then plated onto Petri dishes containing PDA or R2A medium. After three days of incubation at 23–25 °C, colonies that formed were isolated and transferred to fresh sterile plates for further cultivation [48].

DNA from a single colony of bacteria and fungi was extracted using the hexadecyltrimethylammonium bromide (CTAB) method with some modifications [49]. Bacterial 16S rRNA gene sequences were amplified using universal bacterial primers (8F, 5′AGA GTT TGA TCM TGG CTC AG and 1522R, 5′AAG GAG GTG ATC CAR CCG CA) to produce approximately 1500 bp PCR products [50]. For identification of endophytic fungi, the universal primers (5′AGG AGA AGT CGT AAC AAG G and 5′TCC TCC GCT TAT TGA TAT GC) were used to amplify approximately 580 bp ITS1 PCR products. The PCR products were then sequenced on an ABI 3130 Genetic Analyzer (Applied Biosystems, Foster City, CA, USA) and analyzed using the BLAST program. Sequence analysis was performed by multiple sequence alignment using the Clustal X program [51], with a sequence identity of ≥99% used as the threshold for taxonomic identification (Supplementary Material S1).

### 2.2. Seed Germination of A. thaliana in the Presence of Grapevine Endophytes

For experiments, we used seeds of *A. thaliana* ecotype Columbia-0, stored by our lab. Seeds were surface-sterilized by exposure to chlorine vapor generated by adding 3 mL of concentrated HCl to 100 mL of 7% bleach solution from Sayansk Himplast (Sayansk, Russia) for 40–50 min. Following sterilization, seeds were germinated on ^1^/_2_ Murashige and Skoog medium (MS) with a pH of 7.0, solidified with 0.8% (m/v) agar, in a controlled environment chamber at 22 °C under a 16 h light/8 h dark photoperiod with a light intensity of 120 μmol m^−2^ s^−1^. Plants grown under these conditions were used as a negative control.

The following procedures were performed for inoculation with grapevine endophytes. The single colony of bacteria was placed in 20 mL of a liquid nutrient medium, R2A medium (PanReac, AppliChem, Darmstadt, Germany), and incubated for 24–48 h at 28 °C at 150 rpm on an orbital shaker BioSan ES 20/60 (Riga, Latvia), raising the final concentration of 10^7^ colony-forming units per ml (CFU/mL). Next, 100 µL of each bacterial isolate was surface distributed by spreading with a sterilized loop on Petri dishes (diameter 12 cm) with MS medium. Fungal isolates were cultured on a Potato Dextrose Agar (PDA, Neogene, UK), and then, 1 cm^2^ pieces of mycelium and spores were placed on Petri dishes with MS medium. Previous studies on optimizing the concentration of bacteria and fungi have shown that these concentrations are optimal. As lower concentrations of bacteria or a smaller area of inoculated fungus have no effect on plant growth, while higher ones greatly suppress it, the plants did not survive after inoculation.

Under aseptic conditions, *Arabidopsis* seeds sterilized with chlorine vapor were sown on the MS medium in Petri dishes inoculated with endophytes. After 7–8 days, plant endophytic interactions in vitro, the length of the root and height of the stem of the *A. thaliana* seedlings were measured using a ruler (Supplementary Figures S1–S4). Subsequently, seedlings were transferred into pots filled with commercially available rich soil (“Universalniy”, Fasko, Moscow, Russia) in an environmental sterile control chamber (Sanyo MLR-352, Panasonic, Tokyo, Japan) kept on a 16/8 h light/darkness cycle at 22 °C and a light intensity of 120 μmol m^−2^s^−1^.

After one month, the rosette diameter and the number of leaves of the *A. thaliana* plants were measured. In addition, the approximate number of *Arabidopsis* seeds per 1 plant was calculated at the end of the seed ripening period. Dried pods were collected from each plant that had germinated with either grapevine-derived endophyte. The seeds were then removed from the pod, and the total weight of seeds per plant was weighed. Subsequently, 100 seeds from each plant were counted and weighed separately. The total number of seeds per plant was then calculated by dividing the total seed mass by the average mass of 100 seeds. Statistical analyses were performed to determine the mean seed number per plant and the standard error of the mean for each endophyte treatment.

### 2.3. Re-Isolation of the Endophytes That Positively Affect the Growth of Arabidopsis Plants

The endophytic content of bacteria *B. velezensis*, *G. aichiensis* and *Sphingomonas* sp., as well as fungi *Xylaria* sp., *Didymella* sp. and *Exobasidium* sp., was evaluated by counting CFUs of microorganisms in seedling tissue, including leaves, stems and roots, 7 days after inoculation of *Arabidopsis* seeds with endophytes, as described above [52]. For CFU estimation, 10 mg samples of each individual experimental seedling were superficially sterilized in the following order: 75% ethanol for 1 min, 10% H_2_O_2_ for 3 min and distilled water. The samples were homogenized in a sterile mortar using a pestle, with 1 mL of sterile water added. Two consecutive 10-fold dilutions of the resulting homogenate were performed. Aliquots of 100 µL were spread over the surfaces of R2A and PDA media using a microbiological spatula, until they were completely dry. Petri dishes were then incubated in the dark at 28 °C for 48 h. CFU were counted for the second and third dilutions, and their number was recalculated per 1 g of plant wet weight.

### 2.4. RNA Extraction and qRT-PCR

RNA extraction utilized the CTAB-based method outlined by Kiselev et al. [53]. cDNAs were synthesized using the MMLV Reverse Transcription PCR Kit with Oligo(dT)15 (RT-PCR, Evrogen, Moscow, Russia) following Aleynova et al. [54].

Three genes governing auxin metabolism (*AtNIT1*, *AtTAA1*, and *AtYUCCA1*) [55,56], four genes regulating cytokinin metabolism (*AtCYP735A2*, *AtUGT76C2*, *AtCKX4*, and *AtCKX5*) [57,58,59], two genes involved in gibberellin metabolism (*AtGA3ox2* and *AtGA2ox2*) [60,61], four genes associated with abscisic acid metabolism (*AtNCED3* and *AtABA3*) [62], and two genes implicated in ethylene metabolism (*AtEIN2* and *AtEIN3*) [63,64] were analyzed. mRNA transcript levels were assessed via quantitative real-time PCR (qRT-PCR), employing *AtGAPDH* (NM_111283.4), *AtEF1a* (XM_002864638), *AtUBQ* (NM_001084884), and *AtUBC* (XM_021022221) as internal controls. qRT-PCR was performed using separate amplification reactions for the gene of interest and each reference gene. Relative gene expression levels were calculated using the 2^−ΔΔCt^ method [65], with the transcriptional level in the negative control (NC)—uninoculated *A. thaliana* plants—normalized to a value of 1. To ensure robustness, expression values were averaged across multiple technical replicates, and statistical analysis was conducted to assess significance. Data are presented as mean relative expression ± standard error, derived from independent qRT-PCR using multiple validated reference, as per Aleynova et al. [66]. The functions of the selected genes and the sequence of the primers for qRT-PCR are described in a previously published paper [67] and are also provided in Table 1.

qRT-PCR reactions were conducted in 20 µL volumes with the Real-Time PCR Kit (Evrogen, Moscow, Russia) according to Aleynova et al. [54], comprising 1× Taq buffer, 2.5 mM MgCl_2_, 0.2 mM of each dNTP, 0.2 µM of each oligonucleotide primer, 1× SYBR Green I real-time PCR dye, 1 µL cDNA, and 1 unit of Taq DNA polymerase (Evrogen, Moscow, Russia). Analysis was performed using a DTprime 4M1 thermal cycler (DNA-Technology, Moscow, Russia) programmed for an initial denaturation step of 2 min at 95 °C, followed by 50 cycles of 10 s at 95 °C and 25 s at 62 °C.

### 2.5. Statistical Analysis

During our plant experiments, we conducted five independent trials, each comprising 36 plants per treatment. In addition, we carried out two independent experiments for qRT-PCR to confirm our findings, with a total of 8 technical replicates—two for each of the following reference genes: *AtGAPDH*, *AtEF1a*, *AtUBQ*, and *AtUBC*. All data are presented as mean ± standard error (SE) and analyzed by the ANOVA with Tukey post-test and Student’s *t*-test. Correlation analysis was conducted in Excel (Microsoft Office, 2019, Washington, DC, USA) using the appropriate function.

## 3. Results

### 3.1. The Identification of the Most Prevalent Grapevine Endophytes

As a result of the analysis of the collection of endophytic bacteria and fungi of *V. amurensis* grapes, we have selected the most common species of endophytes of wild grapevines. These are bacteria *Agrobacterium rubi* (MZ424738, 99%), *Bacillus velezensis* (CP140115.1, 100%), *Curtobacterium flaccumfaciens* (MZ424740, 100%), *Erwinia billingiae* (KM408608.1, 100%), *Gordonia aichiensis* (BioProject PRJNA1267753, 100%), *Pantoea agglomerans* (MT605813.1, 99%), *Pseudomonas alkylphenolica* (MZ424743, 99%), *Sphingomonas aerolata* (PX909750, 99%), and *Xanthomonas campestris* (MZ424744, 99%), and fungi *Biscogniauxia maritima* (MZ427923, 100%), *Cladosporium perangustum* (MZ427924, 100%), *Didymella pinodella* (MZ427926, 100%), *Exobasidium japonicum* (PX916210, 96%), *Penicillium brevicompactum* (PX916211, 96%), *Pestalotiopsis biciliate* (PX916212, 99%) and *Xylaria flabelliformis* (PX920275, 100%) (Table 2, Supplementary Material S1). It is important to note that using whole-genome sequencing, we were able to identify two bacterial isolates to the species level: *Bacillus* sp. as *B. velezensis* (CP140115.1) [70] and *Gordonia* sp. as *G. aichiensis* (PRJNA1267753) [71].

### 3.2. Growth Characteristics of A. thaliana Plants Inoculated with Grapevine Endophytes

**Figure 1:**
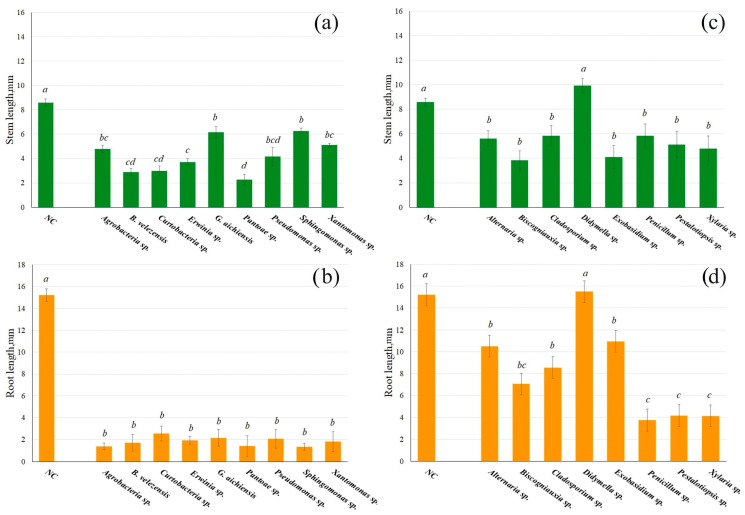
The stem (**a**,**c**) (green color) and root (**b**,**d**) (orange color) lengths of 7-day-old *Arabidopsis thaliana* seedlings were measured after being grown on a ½ Murashige and Skoog (MS) nutrient medium with endophytic bacteria (**a**,**b**) and fungi (**c**,**d**) from wild grapevine *Vitis amurensis* distributed on the surface of the MS medium. The negative control (NC)—uninoculated *A. thaliana* seedlings. Data represent mean ± SE from five independent experiments with twenty technical replicates. Mean values that are followed by the same letter did not differ according to the ANOVA with Tukey post-test. The accepted significance level was *p* < 0.05.

The grapevine endophytes were distributed onto the surface of Petri dishes containing MS medium, where sterile *Arabidopsis* seeds were then placed. After 7 days of inoculation of *A. thaliana* seeds with grapevine endophytic bacteria, a significant delay in seedling development was observed, affecting both shoot (stem) and root growth. The *Arabidopsis* root appears 2–3 days after exposure to a nutrient medium under normal conditions. In the case of seeds treated with grapevine endophytes, the root appeared on 4–6 days. The length of the stem was 1.1–2.9 times smaller when germinated with endophytic bacteria, and the root was 2.1–11.1 times smaller compared to control plants (Figure 1a,b). Seedling development was most strongly inhibited by bacteria of the genus *Pantoea*.

The inhibition of *A. thaliana* seedling development, inoculated with endophytic grapevine fungi, was not as strong as compared to seed germination in the presence of endophytic bacteria (Figure 1c,d). In general, the stem was 1.5–2.3 times smaller and the root 1.4–4.5 times smaller. An exception was the treatment with *Didymella* sp., which did not significantly change the size of *A. thaliana* seedlings (Figure 1c,d).

**Figure 2:**
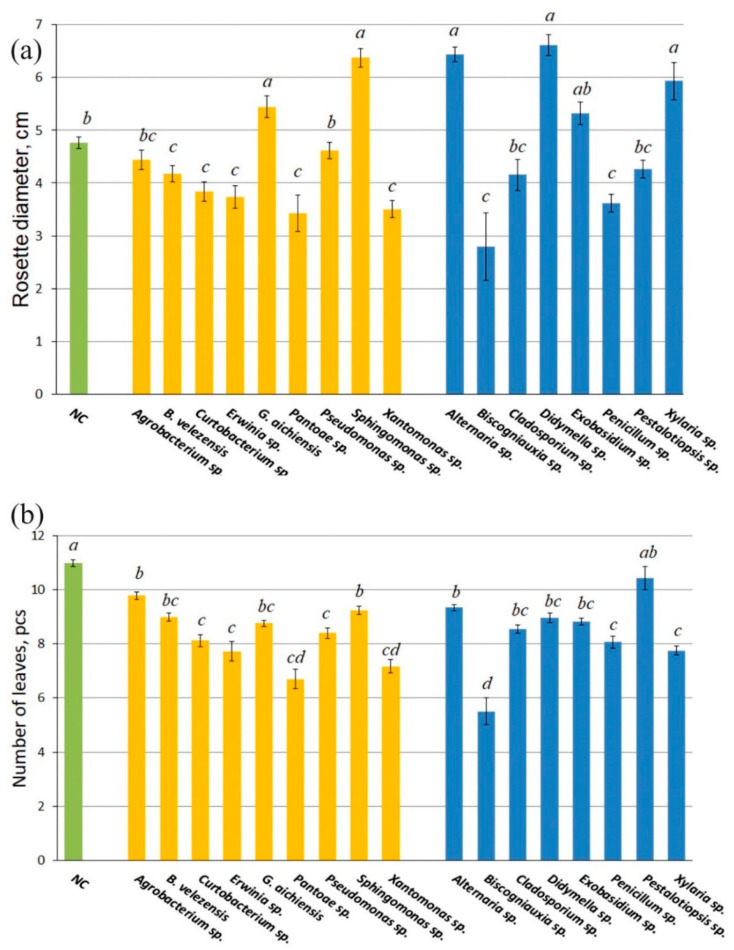
The rosette diameter (**a**) and leaf number (**b**) of 1-month-old *Arabidopsis thaliana* plants growing in the pots with soil after inoculation with endophytic bacteria (yellow color) and fungi (blue color). The negative control (NC)—uninoculated *A. thaliana* plants. Data represent mean ± SE from five independent experiments with twenty technical replicates. Mean values that are followed by the same letter did not differ according to the ANOVA with Tukey post-test. The accepted significance level was *p* < 0.05.

After inoculation with endophytes on a MS medium (contains sucrose as a carbon source) in Petri dishes, *Arabidopsis* seedlings were planted in non-sterile, commercially available, rich soil and cultivated in a climate chamber for 30 days. When *A. thaliana* plants were further cultured, different effects on rosette diameter, number of leaves, and fresh weight accumulation were observed. The diameters of the rosettes of 1-month-old *A. thaliana* plants grown in the presence of the endophytic bacteria *Gordonia aichiensis* and *Sphingomonas* sp. and the fungi *Didymella* spp., *Exobasidium* sp., and *Xylaria* sp. were 1.2–1.4 times larger than those of the control plants (Figure 2a). The diameter of rosettes of *A. thaliana* plants grown in the presence of endophytic bacteria *Bacillus velezensis*, *Curtobacterium* sp., *Erwinia* sp., *Pantoea* sp., *Pseudomonas* sp., and *Xanthomonas* sp., and fungi *Biscogniauxia* sp. and *Penicillium* sp., was significantly smaller compared to the control plants. The number of leaves of 1-month-old plants grown in the presence of wild grapevine endophytes was significantly reduced, except for plants germinated together with the fungus *Pestalotiopsis* sp. (Figure 2b).

Next, three endophytic bacterial isolates (*B. velezensis*, *G. aichiensis*, and *Sphingomonas* sp.) and three fungal isolates (*Xylaria* sp., *Didymella* sp. and *Exobasidium* sp.) were selected based on their strongest positive effects on the rosette diameter and leaf number. Their impact on the accumulation of fresh weight of *Arabidopsis* plants was then investigated in detail.

As a result of re-isolation of endophytes from leaves, stems and roots of 7-day-old *A. thaliana* seedlings, it was found that tissues contained bacteria *B. velezensis*, *G. aichiensis* and *Sphingomonas* sp. in the amounts of 330 × 10^3^, 270 × 10^3^ and 300 × 10^3^ CFU/g of wet weight, respectively. The CFU numbers of *Xylaria* sp., *Didymella* sp. and *Exobasidium* sp. were 20–50 × 10^3^ CFU/g of wet weight.

**Figure 3:**
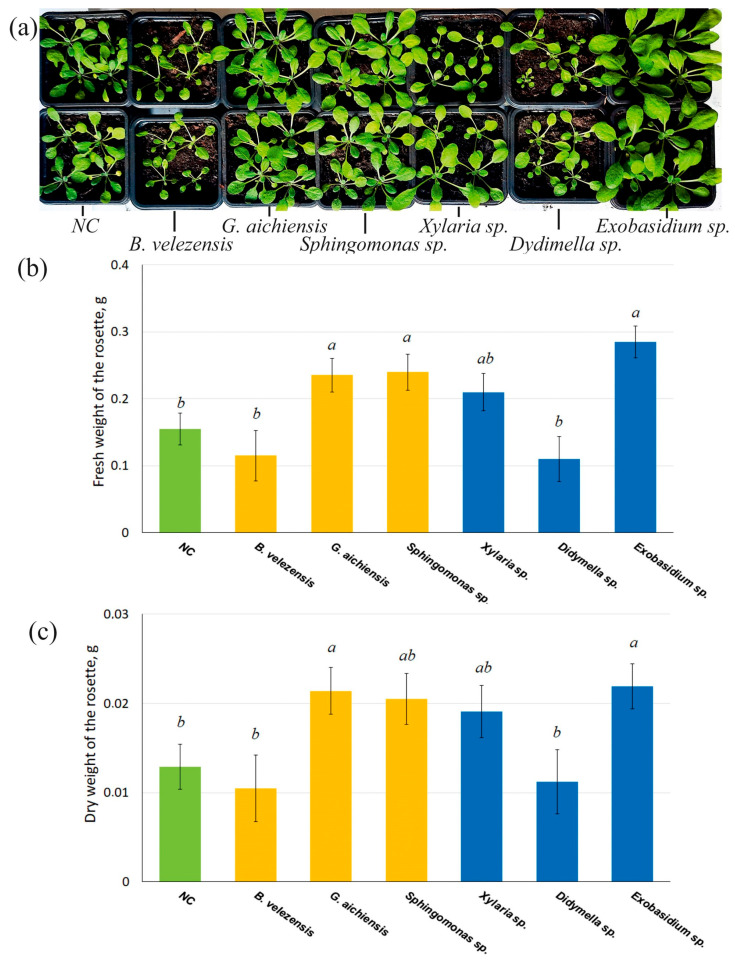
The aerial growth (**a**), fresh (**b**) and dry (**c**) weight accumulation of 1-month-old *Arabidopsis thaliana* plants after inoculation with endophytic bacteria (yellow) and fungi (blue). The negative control (NC, green)—uninoculated *Arabidopsis* plants. Data represent mean ± SE from five independent experiments with twenty technical replicates. Mean values that are followed by the same letter did not differ according to the ANOVA with Tukey post-test. The accepted significance level was *p* < 0.05.

Treatment with *G. aichiensis*, *Sphingomonas* sp., and *Exobasidium* sp. significantly increased the accumulation of fresh and dry weight in *Arabidopsis* rosettes by 1.5–1.8 times (Figure 3), compared to untreated controls. Thus, it was these endophytic microorganisms that had the best effect. The remaining isolates had no significant effect on the accumulation of fresh weight in *Arabidopsis* plants (Figure 3).

**Figure 4:**
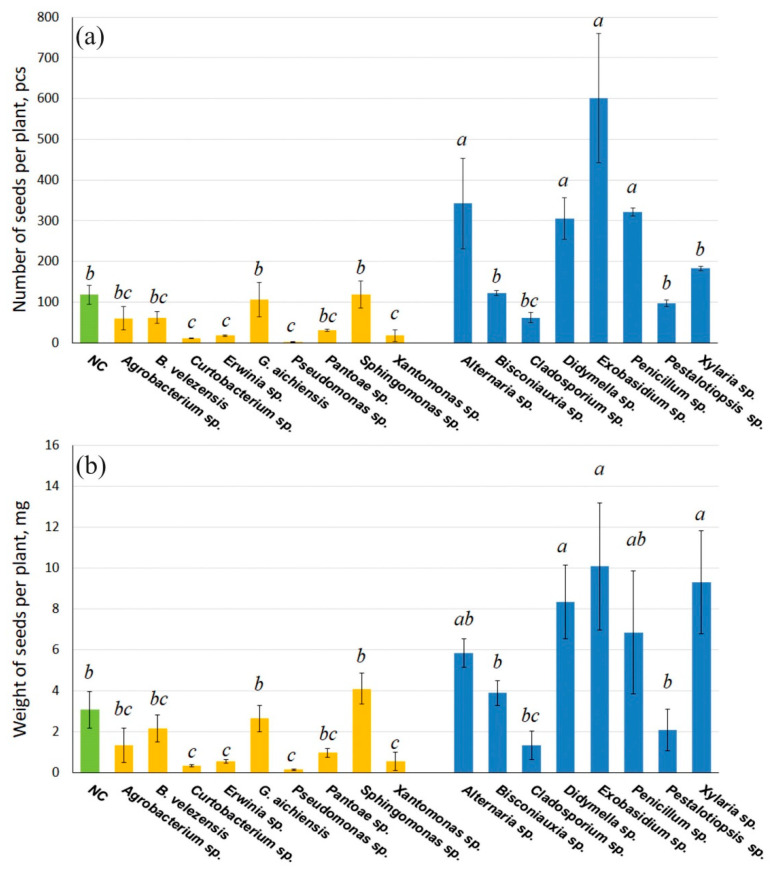
The number (**a**) and weight (**b**) of seeds per one *Arabidopsis thaliana* plant after inoculation with endophytic bacteria (yellow) and fungi (blue) from wild grapevine *Vitis amurensis*. The negative control (NC, green)—uninoculated *Arabidopsis* plants. Data represent mean ± SE from five independent experiments with twenty technical replicates. Mean values that are followed by the same letter did not differ according to the ANOVA with Tukey post-test. The accepted significance level was *p* < 0.05.

After the formation and maturation of *A. thaliana* pods, seeds were collected and counted from each group of plants grown in the presence of selected endophytic bacteria and fungi. Wild-type *A. thaliana* plants grown without exposure to *V. amurensis*-derived endophytic microorganisms produced an average of 100 seeds per plant, weighing 3 mg (Figure 4). Seed production was significantly reduced by 6.5–38-fold when *A. thaliana* was inoculated with the grape endophytic bacteria *Erwinia* sp., *Pantoea* sp., *Pseudomonas* sp. and *Xanthomonas* sp. The minimum number of seeds was observed when germinating together with endophytic bacteria of the genus *Pseudomonas* and amounted to three seeds per plant. The bacteria *Agrobacterium* sp., *B. velezensis*, *G.aichiensis*, and *Sphingomonas* sp. did not significantly affect the production of *Arabidopsis* seeds (Figure 4).

Inoculation of *A. thaliana* seeds with endophytic fungi of wild grape *V. amurensis* did not reduce seed production and weight. In addition, inoculation of seeds in the presence of the fungi *Xylaria* sp., *Didymella* sp., *Exobasidium* sp., and *Penicillium* sp. significantly increased seed production and weight by 2.5–5 times. The highest seed production was observed in plants germinated together with *Exobasidium* sp. (Figure 4). Thus, the fungus *Exobasidium* sp. enhances the size and yield of *Arabidopsis* more effectively than other grapevine endophytes studied.

### 3.3. Gene Expression of Phytohormone Metabolism Genes in Arabidopsis Plants After Inoculation with V. amurensis Endophytic Microorganisms

**Figure 5:**
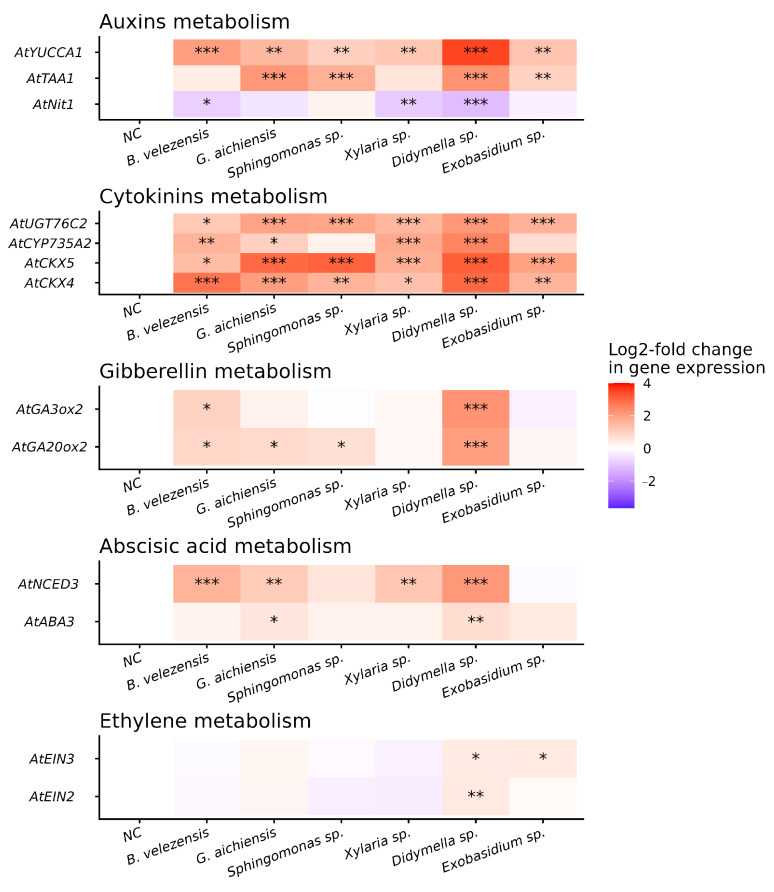
Heatmap of the phytohormone metabolism genes *AtNIT1*, *AtTAA1*, *AtYUCCA1*, *AtCKX4*, *5*, *AtCYP735A2*, *AtUGT76C2*, *AtGA3ox2*, *AtGA2ox2*, *AtNCED3*, *AtABA3*, and *AtEIN2; 3* expression levels in *Arabidopsis thaliana* 7-day-old seedlings after inoculation with endophytic bacteria and fungi from wild grapevine *Vitis amurensis*. The negative control (NC)—uninoculated *Arabidopsis* plants. Data represent mean ± SE from two independent experiments. *, **, ***—significantly different from the values of gene expression in *A. thaliana* 7-day-old seedling plant under the control conditions (Wt) at *p* ≤ 0.05, 0.01 and 0.001 according to Student’s *t*-test.

In order to explain the effects of grapevine endophytes on plant growth, we analyze the expression level of *A. thaliana* genes encoding phytohormone biosynthesis in leaves, stems and roots of 7-day-old seedlings. The expression of seedlings inoculated with bacteria and fungi was analyzed, which demonstrated an increase in plant size and biomass by 1 month, particularly after inoculation with endophytic bacteria *B. velezensis*, *G. aichiensis*, and *Sphingomonas* sp. and fungi *Xylaria* sp., *Didymella* sp., and *Exobasidium* sp. (Figure 5; Table 2). The qPCR analysis revealed that the level of gene transcription of auxin (*AtTAA1* and *AtYUCCA1*) and cytokinin (*AtCKX4*, *5*, *AtCYP735A2*, and *AtUGT76C2*) metabolism considerably increased in all *Arabidopsis* 7-day-old seedlings inoculated with grapevine endophytes (Figure 5). AtTAA1 and AtYUCCA1 catalyze the first and second stages of auxin synthesis (IAA) via indole-3-pyruvate (IPA). AtCKX4 and AtUGT76C2 are responsible for regulating the content of endogenous CKs. *AtCYP735A2* is the enzyme that catalyzes the synthesis of CKs (Table 2). 

The expression of the *AtNIT1* gene, which is an enzyme that converts indole-3-acetonitrile to IAA, was significantly reduced compared to the control in all *A. thaliana* seedlings inoculated with endophytes. Additionally, the expression of genes involved in ABA (*AtNCED3*, which is an enzyme that catalyzes the rate-limiting step of ABA biosynthesis) and GAs (*AtGA2ox2*, which catalyzes oxidation at a late stage of GA biosynthesis) metabolism was significantly upregulated; a significant increase in all genes of these groups was observed when *A. thaliana* seeds were inoculated with *Didymella* sp. (Figure 5). Furthermore, genes *AtEIN2* and *AtEIN3*, which play key roles in ethylene signaling, showed significantly higher expression in *Arabidopsis* seedlings inoculated with *Didymella* sp. or *Exobasidium* sp. (Figure 5).

We performed the Pearson correlation analysis between the expression levels of genes involved in the phytohormone metabolism and the diameter and mass of 1-month-old *Arabidopsis* rosettes (Supplementary Table S1). Only correlations with an absolute Pearson coefficient (r) greater than 0.5 or less than −0.5 were considered significant. The analysis revealed a selective moderate positive correlation between the expression of genes involved in cytokinin and auxin metabolism and plant size. Specifically, expression of the *AtNIT1* was positively correlated with increased rosette mass (r = 0.591, *p* = 0.001), while expression of *AtTAA1*, *AtCKX5*, and *AtUGT76C2* showed positive correlations with increased *Arabidopsis* rosette diameter (r = 0.654, *p* = 0.002; 0.766, *p* = 0.003; and 0.769, *p* = 0.007) (Supplementary Table S1). In contrast, negative correlations were observed between *Arabidopsis* biomass accumulation and the expression of *AtNCED3* (r = −0.715, *p* = 0.003) involved in ABA metabolism (Supplementary Table S1).

## 4. Discussion

Endophytic microbes enhance plant resistance to both abiotic and biotic stresses [72,73] and can influence seed germination and seedling vigor, with significant implications for plant health and ecosystem dynamics [74]. Despite this potential, studies examining the specific effects of individual endophytic microorganisms on agricultural crops remain limited [75].

The medicinal plants, which include wild grapevine *V. amurensis*, could be a promising source for isolating plant-beneficial endophytes that can be used to enhance plant growth and protect plants from soil-borne pathogens [31,32,48]. This paper analyzes the influence of the most frequently encountered endophytic bacteria and fungi of wild grapevine on the growth characteristics of the model plant *A. thaliana*. For 7-day-old *Arabidopsis* seedlings inoculated with endophytic microorganisms from *V. amurensis*, there was a significant inhibition of development, except for seeds inoculated with *Didymella* sp. Significant increases in plant size were observed in one-month-old seedlings inoculated with the endophytic bacteria *G. aichiensis* and *Sphingomonas* sp., and the fungi *Xylaria* sp., *Didymella* sp., and *Exobasidium* sp. Thus, the effect of endophytes on *A. thaliana* plants is contingent upon the specific endophyte species and its strategy of interaction with the host. Initially, seedlings exposed to high microbial concentrations appear to activate canonical defense responses, such as upregulation of genes involved in ABA metabolism, a hormone typically accumulated under stress conditions [76], or delayed seed development may stem from microbial metabolites [77] or germination-inhibiting enzymes [78]. Subsequently, a selective interaction dynamic emerges between the plant and its microbial colonizers, leading to a functional dichotomy: a subset of endophytes act as plant growth promoters, while others behave as conditional pathogens, as evidenced by their negative impact on biomass accumulation and seed production.

Endophytes that have a positive effect on plant size likely accelerate plant development not only through direct synthesis of phytohormones but also by triggering a cascade of host plant hormone biosynthesis gene expression [17,18]. Our results reveal a significant upregulation of genes associated with auxin and CK biosynthesis in *A. thaliana* seedlings colonized by grapevine endophytes. A moderate, though statistically non-significant, trend toward increased expression of ABA- and GA-related genes was also observed. Activation of the genes involved in the metabolism of these important plant hormones is likely to lead to an increase in the diameter and weight of the *Arabidopsis* plants [10,11,79]. Pearson’s correlation analysis revealed a selective positive relationship between the expression of genes involved in CK and auxin biosynthesis and biomass accumulation and plant diameter in *Arabidopsis* rosettes. There was a significant decrease in *AtNIT1* expression in all *Arabidopsis* seedlings inoculated with grapevine endophytes, which had a positive effect on plant size. It is known that *AtNIT1* homologs regulate cell proliferation and differentiation [80] and participate in glucosinolate catabolism [63], which is important for the development of seedlings. Also, the *AtNIT2*, *AtNIT3*, and *AtNIT4* genes were distinctly induced in *A. thaliana* leaves by *P. syringae* pv. *tomato* infection [64]. The observed significant downregulation of *AtNIT1* expression may result from transcriptional repression mediated by the competitive activation of alternative nitrilase isoforms, such as *AtNIT2*, *AtNIT3*, and *AtNIT4*, in response to colonization by grapevine-associated endophytes. This isoform switching likely alters substrate partitioning within the nitrilase metabolic network, thereby reducing the availability of common substrates (e.g., indole-3-acetonitrile) for *AtNIT1*, which may further reinforce its transcriptional suppression through feedback mechanisms. A negative correlation was found between *Arabidopsis* biomass accumulation and the expression of genes involved in GA and ABA metabolism. Also, there could be inhibitory mechanisms outside of hormonal changes, too. It is possible that low biomass accumulation in plants inoculated with *B. velezensis* and *Didymella* sp. is due to activation of *Arabidopsis* protective responses, which eventually leads to a slowdown of active growth and development. It is worth noting that changes in the expression of genes involved in plant hormone metabolism were detected in 7-day-old seedlings, but the growth-stimulating effect manifested later. This delay likely reflects the complex regulation of the hormonal system during plant development: gene expression changes represent an early signaling event, whereas alterations in phytohormone levels require additional time for biosynthesis, transport, and signaling cascade activation [81]. Thus, the expression of plant hormone metabolism genes is usually measured in the early stages of plant development, because after a certain level of phytohormones is accumulated, their expression changes significantly. Therefore, we can assume that endophytic bacteria and fungi of the grapevine *V. amurensis* stimulate the growth of *Arabidopsis* plants by activating different genes involved in phytohormone biosynthesis.

The results demonstrated that the inoculation with most endophytic bacteria significantly reduced the total seed yield of *A. thaliana* seeds. However, this trend was not observed in strains of *G. aichiensis* and *Sphingomonas* sp., which had no significant effect on seed production. While most of the tested endophytic fungi did not significantly affect the seed yield of *A. thaliana* plants, the *Xylaria* sp., *Didymella* sp. and *Exobasidium* sp. increased seed production by 2.5- to 5-fold. This pronounced enhancement correlated with upregulated expression of the ethylene signaling genes *AtEIN2* and *AtEIN3* in *A. thaliana* plants inoculated with *Didymella* sp. and *Exobasidium* sp. *AtEIN2* and *AtEIN3* play distinct but complementary roles in the ET signaling pathway, both being critical for regulating plant growth, development, and stress responses [68,69]. *AtEIN2* serves as a central component in the ethylene signaling cascade, which is involved in both the initial and sustained phases of ET-mediated growth inhibition [82]. *AtEIN3*, a key transcription factor, receives signals relayed by the EIN2-mediated pathway and activates transcriptional reprogramming of ethylene-responsive genes, thereby amplifying the ethylene signal [83].

The observed upregulation of these genes suggests a potential mechanistic link between fungal inoculation, enhanced ethylene signaling, and increased seed yield. However, whether this upregulation directly causes the observed phenotypic effect—or is a correlative response—remains to be determined. Further functional studies are needed to establish causality and elucidate the precise molecular mechanisms underlying this phenomenon.

In addition to activating genes that encode enzymes for hormone metabolism in *Arabidopsis*, plant growth and yield can also occur due to the production of metabolites by endophytes. We have demonstrated that bacteria *B. velezensis* AMR25 and *G. aichiensis* P6PL2 possessed genes for the production of phytohormones (auxins and CKs) and an increased bioavailability of nutrients such as nitrogen, phosphorus, potassium, and sulfur [32,42]. In the future, it is planned to conduct whole-genome sequencing of wild grapevine endophytic microorganisms (*Sphingomonas* sp., *Xylaria* sp., *Didymella* sp., and *Exobasidium* sp.) in order to identify the molecular mechanisms that stimulate the active growth of the host plant.

## 5. Conclusions

The inoculation of dominant endophytic bacteria and fungi from wild grapevine *V. amurensis* exerted differential effects on plant size and seed germination in *A. thaliana*, in part correlating with transcriptional changes in individual genes of hormone biosynthetic pathways. Therefore, a very important aspect in the introduction of endophytic microorganisms into agriculture involves not only the verification of endophyte protective properties (e.g., antipathogen properties or effects on abiotic stress tolerance) but also assessing their impact on crop growth parameters and yield potential.

## Figures and Tables

**Table 1:** Primers for real-time quantitative PCR (qRT-PCR) of the expression of some *Arabidopsis thaliana* genes involved in the regulation of phytohormone metabolism.

#	Gene (GeneBank)	Phytohormone	Gene Functions	Primers	References
1	Nitrilase 1, *AtNIT1* (NM_180680.3)	Auxin metabolism	Converts indole-3-acetonitrile (IAN) into the major plant growth hormone, indole-3-acetic acid (IAA).	5′GGC GTT CAT AAC GAA GAA GGG CGT G, 5′TTC CTT CTC TAT GGC TCC CAT TAC C	[55]
2	Trypthophan amonotransferase of *Arabidopsis* 1, *AtTAA1* (NM_105724.3)	Auxin metabolism	TAA1 belongs to TAA1-Related (TAA1/TAR) family of Trp aminotransferases. It catalyzes the first stage of auxin synthesis (IAA) by transferring the Trp to an alpha-keto acid, and to generate IPyA and another amino acid like L-glutamate.	5′AAC GCT GCG ACG GAG GAT CG, 5′CGT GGA CGG CGG CTT GAC AA	[56]
3	Flavin monooxygenases, *AtYUCCA1* (XP_002869265.2)	Auxin metabolism	Participates in the second step of auxin synthesis (IAA) by converting IPyA into IAA via an oxygen and NADPH-dependent reaction.	5′TCC GCA TCG CTC CAA GGT TC, 5′GGA AGT ATG GAT CTG CGT TCT CAC C	[56]
4	Cytokinin oxidase 4, *AtCKX4* (NM_001341977)	Cytokinins metabolism	CKX is responsible for regulating the content of endogenous CKs by oxidative side chain removal. This enzyme catalyzes the catabolism of specific CKs to inactive products that lack the N6-unsaturated side chain.	5′TGG GTG GAT GTT CTG AAG GCG, 5′ACG TTA CTA ATC TGA GGG CCG T;	[57]
5	Cytokinin oxidase 5, *AtCKX5* (NM_106199.5)	Cytokinins metabolism	CKX is responsible for regulating the content of endogenous CKs by oxidative side chain removal. This enzyme catalyzes the catabolism of specific CKs to inactive products that lack the N6-unsaturated side chain	5′GAG CCA TTG GCC GTG CTT CA, 5′AAC CAC CAC ACC GTT CCT CCC	[57]
6	Cytochrome P450 monooxygenase, *AtCYP735A2* (NM_105381.5)	Cytokinins metabolism	Encode cytokinin hydroxylases that catalyze the biosynthesis of *trans*-zeatin.	5′CTA AAC CCC GTC TCC TCA CC, 5′CTC TTC CCA TAT TGT TTG GAC C	[58]
7	Cytokinin N-glucosyltransferase, *AtUGT76C2* (NM_120668.4)	Cytokinins metabolism	Cytokinin glycosyltransferase, which is involved in the regulation of CK homeostasis in plants.	5′CCA TTA CCG TGA TCC ACA CG, 5′CAC GAA ACG GAG ACT CAG CG	[59]
8	Gibberellin 3 beta-hydroxylase, *AtGA3ox2* (NM_106683.2)	Gibberellin metabolism	Responsible for converting inactive GAs into biologically active ones.	5′CTG CCG CTC ATC GAC CTC, 5′AGC ATG GCC CAC AAG AGT G	[60]
9	Gibberellin 20-oxidase, *AtGA20ox2* (NM_124560.4)	Gibberellin metabolism	Catalyze consecutive steps of oxidation in the late part of the GA biosynthetic pathway.	5′AGA AAC CTT CCA TTG ACA TTC CA, 5′AGA GAT CGA TGA ACG GGA CG	[61]
10	9-cis-epoxycarotenoid dioxygenase, *AtNCED3* (NM_112304.3)	Abscisic acid metabolism	The enzyme catalyzes the rate-limiting step of ABA biosynthesis.	5′AGCTAACCCACTTCACGAGC, 5′CCAATTGACGTTCCTGAAC	[62]
11	Molybdenum cofactor sulfurase, *AtABA3* (NM_001332230)	Abscisic acid metabolism	Participating in the activation of aldehyde oxidase and xanthine dehydrogenase. These enzymes are involved in ABA biosynthesis and in purine degradation.	5′TCACATCATTGGGCGGTTGT, 5′AGATCTTTCCCTTTACTCTC	[62]
12	Ethylene-insensitive 2 transmembrane protein, *AtEIN2* (AF141202)	Ethylene metabolism	A protein that is involved in the ET signaling pathway in plants. It is an integral membrane protein located in the endoplasmic reticulum.	5′GAGAGTCGGCCTGAGCTTTG, 5′GTGGCTCGCTGGAATCTGA	[68]
13	Ethylene-insensitive 3 transcription factor, *AtEIN3* (NM_112968)	Ethylene metabolism	A key transcription factor in the ET signaling pathway in plants.	5′ACAGTAGCGGCAACAGGTTC, 5′TTGCTGCTTCTGCTGCATTC	[68,69]

**Table 2:** The identity of the bacterial and fungal isolates obtained from the *Vitis amurensis* microbiome, based on the analysis of 16S rRNA and ITS1 gene sequences for bacteria and fungi. The resulting nucleotide sequences were generated using the Staden Package software 1.6-r. The similarity of the collected nucleotide sequences was assessed using the NCBI BLAST tool (http://blast.ncbi.nlm.nih.gov/; accessed on 21 January 2026), employing the nucleotide-nucleotide BLAST algorithm v.2.17.0.

№	Used Gene	Genus and Sequence ID	The Close Species and Sequence ID	Percent Identity
1	*16S rRNA*	*Agrobacterium* (MZ424738)	*Agrobacterium rubi* (MN752429.1) [48]	99.17%
2	Whole genome	*Bacillus velezensis*	*Bacillus velezensis* (CP140115.1) [70]	100%
3	*16S rRNA*	*Curtobacterium* (MZ424740)	*Curtobacterium flaccumfaciens* (AJ310414.1) [31]	100%
4	*16S rRNA*	*Erwinia* (MZ424741)	*Erwinia billingiae* (KM408608.1) [48]	100%
5	Whole genome	*Gordonia aichiensis*	*Gordonia aichiensis* (BioProject PRJNA1267753) [71]	100%
6	*16S rRNA*	*Pantoea* (MZ424742)	*Pantoea agglomerans* (MT605813.1) [48]	99.75%
7	*16S rRNA*	*Pseudomonas* (MZ424743)	*Pseudomonas alkylphenolica* (MN813762.1) [48]	99.89%
8	*16S rRNA*	*Sphingomonas* (PX909750)	*Sphingomonas aerolata* (CP098762)	99%
9	*16S rRNA*	*Xanthomonas* (MZ424744)	*Xanthomonas campestris* (MN108237.1) [48]	99.13%
10	*ITS1*	*Biscogniauxia* (MZ427923)	*Biscogniauxia maritima* (MN341558.1) [48]	100%
11	*ITS1*	*Cladosporium* (MZ427924)	*Cladosporium perangustum* (MT645918.1) [48]	100%
12	*ITS1*	*Didymella* (MZ427926)	*Didymella pinodella* (KX869956.1) [48]	100%
13	*ITS1*	*Exobasidium* (PX916210)	*Exobasidium japonicum* (EU692773.1)	96%
14	*ITS1*	*Penicillium* (PX916211)	*Penicillium brevicompactum* (MW018697.1)	96%
15	*ITS1*	*Pestalotiopsis* (PX916212)	*Pestalotiopsis biciliate* (PP146582.1)	99%
16	*ITS1*	*Xylaria* (PX920275)	*Xylaria flabelliformis* (PQ632332.1)	100%

## Data Availability

The data presented in this study are available within the article and Supplementary Materials.

## Supplementary Materials

The following are available online at https://www.mdpi.com/article/10.3390/life16040566/s1, Supplementary Table S1. The correlation analysis of phytohormone metabolism gene expression with *Arabidopsis* rosette mass and diameter; Supplementary Material S1. Characteristics of bacteria and fungi based on 16S rRNA (bacteria) or ITS1 (fungi) gene sequences used in experiments isolated from the *Vitis amurensis* grapevine microbiome; Supplementary Figure S1. *Arabidopsis* seed germination with endophytic grapevine bacteria *Vitis amurensis*; Supplementary Figure S2. *Arabidopsis* seed germination with endophytic grapevine fungi *Vitis amurensis*; Supplementary Figure S3. Measurement of root length and stem height of *Arabidopsis* seedlings germinated with *Vitis amurensis* grapevine endophytes; Supplementary Figure S4. Measurement of root length and stem height of *Arabidopsis* seedlings germinated with *Vitis amurensis* grapevine endophytes.

## Abbreviations

The following abbreviations are used in this manuscript:

ACC deaminase	1-aminocyclopropane-1-carboxylate deaminase
CKs	cytokinins
GAs	gibberellins
ABA	abscisic acid
ET	ethylene
