# Supplementary material for: The Influence of Wild Grapevine Endophytes on the Growth of the Model Plant Arabidopsis thaliana (L.) Heynh
Source: Life (Basel). 2026 Mar 31;16(4):566. doi: 10.3390/life16040566 (PMC13117498; doi:10.3390/life16040566)
Supplement: Supplementary file 1 [file life-16-00566-s001.zip › life-4197478-supplementary.pdf]

Supplementary Table S1. The correlation analysis of phytohormone metabolism gene expression with *Arabidopsis* rosette mass and diameter.

**Gene**	**Phytohormone**	**Correlation fresh weight of the rosette with expression**	**Correlation rosette diameter with expression**
Nitrilase 1, *AtNIT1* (NM_180680.3)	Auxin metabolism	**0,591**	0,105
Trypyhophan amonotransferase of Arabidopsis 1, *AtTAA1* (NM_105724.3)	Auxin metabolism	0,021	**0,654**
Flavin monooxygenases, *AtYUCCA1* (XP_002869265.2)	Auxin metabolism	-0,481	0,419
Cytokinin oxidase 4, *AtCKX4* (NM_001341977)	Cytokinins metabolism	-0,419	0,088
Cytokinin oxidase 5, *AtCKX5* (NM_106199.5)	Cytokinins metabolism	0,130	**0,766**
Cytochrome P450 monooxygenase, *AtCYP735A2* (NM_105381.5)	Cytokinins metabolism	-0,492	0,398
Cytokinin N-glucosyltransferase, *AtUGT76C2* (NM_120668.4)	Cytokinins metabolism	0,203	**0,769**
Gibberellin 3 beta-hydroxylase, *AtGA3ox2* (NM_106683.2)	Gibberellin metabolism	-0,391	0,395
Gibberellin 20-oxidase, *AtGA20ox2* (NM_124560.4)	Gibberellin metabolism	**-0,472**	**0,494**
9-cis-epoxycarotenoid dioxygenase, *AtNCED3* (NM_112304.3)	Abscisic acid metabolism	**-0,715**	0,279
Molybdenum cofactor sulfurase, *AtABA3* (NM_001332230)	Abscisic acid metabolism	-0,012	0,346
Ethylene-insensitive 2 transmembrane protein, *AtEIN2* (AF141202)	Ethylene metabolism	-0,302	0,284
Ethylene-insensitive 3 transcription factor, *AtEIN3* (NM_112968)	Ethylene metabolism	0,138	0,222

Supplementary Material S1. The identity of bacteria and fungi based on 16S rRNA (bacteria) or ITS1 (fungi) gene sequences used in experiments isolated from *Vitis amurensis* grapevine microbiome.

The resulting nucleotide sequences were collected using the Staden Package program. The percent identity of the collected nucleotide sequences is determined using a specialized program NCBI BLAST (http://blast.ncbi.nlm.nih.gov), using the Nucleotide Blast (nucleotide - nucleotide BLAST) algorithm.

1. ***Agrobacterium* sp.,** Sequence ID MZ424738, (s1860-1,7,13,19), 1443 bp.

AGAGTTTGATCCTGGCTCAGAACGAACGCTGGCGGCAGGCTTAACACATGCAAGTCGAACGCATCGCAAGATGAGTGGCAGACGGGTGAGTAACGCGTGGGAATCTACCCATCTCT
GCGGAATAGCTCTGGGAAACTGGAATTAATACCGCATACGCCCTACGGGGGAAAGATTTATCGGGGATGGATGAGCCCGCGTTGGATTAGCTAGTTGGTGGGGTAAAGGCCTACCA
AGGCGACGATCCATAGCTGGTCTGAGAGGATGATCAGCCACATTGGGACTGAGACACGGCCCAAACTCCTACGGGAGGCAGCAGTGGGGAATATTGGACAATGGGCGCAAGCCTG
ATCCAGCCATGCCGCGTGAGTGATGAAGGCCTTAGGGTTGTAAAGCTCTTTCACCGGTGAAGATAATGACGGTAACCGGAGAAGAAGCCCCGGCTAACTTCGTGCCAGCAGCCGCGG
TAATACGAAGGGGGCTAGCGTTGTTCGGAATTACTGGGCGTAAAGCGCACGTAGGCGGATATTTAAGTCAGGGGTGAAATCCCGCAGCTCAACTGCGGAACTGCCTTTGATACTGGG
TATCTTGAGTATGGAAGAGGTAAGTGGAATTGCGAGTGTAGAGGTGAAATTCGTAGATATTCGCAGGAACACCAGTGGCGAAGGCGGCTTACTGGTCCATTACTGACGCTGAGGTG
CGAAAGCGTGGGGAGCAAACAGGATTAGATACCCTGGTAGTCCACGCCGTAAACGATGAATGTTAGCCGTCGGGCAGTTGACTGTTCGGTGGCGCAGCTAACGCATTAAACATTCCG

CCTGGGGAGTACGGTCGCAAGATTAAAACTCAAAGGAATTGACGGGGGCCCGCACAAGCGGTGGAGCATGTGGTTTAATTCGAAGCAACGCGCAGAACCTTACCAGCTCTTGACATT
CGGGGTTTGGGCAGTGGAGACATTGTCCTTCAGTTAGGCTGGCCCCAGAACAGGTGCTGCATGGCTGTCGTCAGCTCGTGTCGTGAGATGTTGGGTTAAGTCCCGCAACGAGCGCAA
CCCTCGCCCTTAGTTGCCAGCATTTGGTTGGGCACTCTAAGGGGACTGCCGGTGATAAGCCGAGAGGAAGGTGGGGATGACGTCAAGTCCTCATGGCCCTTACGGGCTGGGCTACAC
ACGTGCTACAATGGTGGTGACAGTGGGCAGCGAGACAGCGATGTCGAGCTAATCTCCAAAAGCCATCTCAGTTCGGATTGCACTCTGCAACTCGAGTGCATGAAGTTGGAATCGCTA
GTAATCGCAGATCAGCATGCTGCGGTGAATACGTTCCCGGGCCTTGTACACACCGCCCGTCACACCATGGGAGTTGGTTTTACCCGAAGGCGCTGCGCTAACCGCAAGGGGGCAGGC
GACCACGGTAGGGTCAGCGACTGGGGTGAAGTCGTAACAAGGTA

***Agrobacterium rubi*,** Sequence ID MN752429.1, 1443bp, Percent identity 99.17%

AGAGTTTGATCATGGCTCAGAACGAACGCTGGCGGCAGGCTTAACACATGCAAGTCGAACGCCCCGCAAGGGGAGTGGCAGACGGGTGAGTAACGCGTGGGAATCTACCCAACCCT
GCGGAATAGCTCTGGGAAACTGGAATTAATACCGCATACGCCCTACGGGGGAAAGATTTATCGGGGATGGATGAGCCCGCGTTGGATTAGCTAGTTGGTGGGGTAAAGGCCTACCA
AGGCGACGATCCATAGCTGGTCTGAGAGGATGATCAGCCACATTGGGACTGAGACACGGCCCAAACTCCTACGGGAGGCAGCAGTGGGGAATATTGGACAATGGGCGCAAGCCTG
ATCCAGCCATGCCGCGTGAGTGATGAAGGCCTTAGGGTTGTAAAGCTCTTTCACCGGTGAAGATAATGACGGTAACCGGAGAAGAAGCCCCGGCTAACTTCGTGCCAGCAGCCGCGG
TAATACGAAGGGGGCTAGCGTTGTTCGGAATTACTGGGCGTAAAGCGCACGTAGGCGGATATTTAAGTCAGGGGTGAAATCCCAGAGCTCAACTCTGGAACTGCCTTTGATACTGGG
TATCTTGAGTATGGAAGAGGTAAGTGGAATTGCGAGTGTAGAGGTGAAATTCGTAGATATTCGCAGGAACACCAGTGGCGAAGGCGGCTTACTGGTCCATTACTGACGCTGAGGTG
CGAAAGCGTGGGGAGCAAACAGGATTAGATACCCTGGTAGTCCACGCCGTAAACGATGAATGTTAGCCGTCGGGCAGTTGACTGTTCGGTGGCGCAGCTAACGCATTAAACATTCCG
CCTGGGGAGTACGGTCGCAAGATTAAAACTCAAAGGAATTGACGGGGGCCCGCACAAGCGGTGGAGCATGTGGTTTAATTCGAAGCAACGCGCAGAACCTTACCAGCTCTTGACATT
CGGGGTTTGGGCAGTGGAGACATTGTCCTTCAGTTAGGCTGGCCCCAGAACAGGTGCTGCATGGCTGTCGTCAGCTCGTGTCGTGAGATGTTGGGTTAAGTCCCGCAACGAGCGCAA
CCCTCGCCCTTAGTTGCCAGCATTTAGTTGGGCACTCTAAGGGGACTGCCGGTGATAAGCCGAGAGGAAGGTGGGGATGACGTCAAGTCCTCATGGCCCTTACGGGCTGGGCTACAC
ACGTGCTACAATGGTGGTGACAGTGGGCAGCGAGACAGCGATGTCGAGCTAATCTCCAAAAGCCATCTCAGTTCGGATTGCACTCTGCAACTCGAGTGCATGAAGTTGGAATCGCTA
GTAATCGCAGATCAGCATGCTGCGGTGAATACGTTCCCGGGCCTTGTACACACCGCCCGTCACACCATGGGAGTTGGTTTTACCCGAAGGCGCTGCGCTAACCGCAAGGGGGCAGGC
GACCACGGTAGGGTCAGCGACTGGGGTGAAGTCGTAACAAGGTA

2. ***Bacillus velezensis* AMR25.** The complete genome has been sequenced and published in GenBank (acc. no CP140115.1).
Ananev, A.A.; Ogneva, Z.V.; Nityagovsky, N.N.; Suprun, A.R.; Kiselev, K.V.; Aleynova, O.A. Whole Genome Sequencing of *Bacillus velezensis* AMR25, an Effective Antagonist Strain against Plant Pathogens. Microorganisms 2024, 12, 1533. https://doi.org/10.3390/microorganisms12081533

3. ***Curtobacterium* sp.,** Sequence ID MZ424740, (s1860-3,9,15,21), 1514 bp.

AGAGTTTGATCCTGGCTCAGGACGAACGCTGGCGGCGTGCTTAACACATGCAAGTCGAACGATGATGCCCAGCTTGCTGGGTGGATTAGTGGCGAACGGGTGAGTAACACGTGAGT
AACCTGCCCCTGACTCTGGGATAAGCGTTGGAAACGACGTCTAATACTGGATATGATCACTGGCCGCATGGTCTGGTGGTGGAAAGATTTTTTGGTTGGGGATGGACTCGCGGCCTAT
CAGCTTGTTGGTGAGGTAATGGCTCACCAAGGCGACGACGGGTAGCCGGCCTGAGAGGGTGACCGGCCACACTGGGACTGAGACACGGCCCAGACTCCTACGGGAGGCAGCAGTG
GGGAATATTGCACAATGGGCGAAAGCCTGATGCAGCAACGCCGCGTGAGGGATGACGGCCTTCGGGTTGTAAACCTCTTTTAGTAGGGAAGAAGCGAAAGTGACGGTACCTGCAGA
AAAAGCACCGGCTAACTACGTGCCAGCAGCCGCGGTAATACGTAGGGTGCAAGCGTTGTCCGGAATTATTGGGCGTAAAGAGCTCGTAGGCGGTTTGTCGCGTCTGCTGTGAAATCC

CGAGGCTCAACCTCGGGCTTGCAGTGGGTACGGGCAGACTAGAGTGCGGTAGGGGAGATTGGAATTCCTGGTGTAGCGGTGGAATGCGCAGATATCAGGAGGAACACCGATGGCG
AAGGCAGATCTCTGGGCCGTAACTGACGCTGAGGAGCGAAAGCATGGGGAGCGAACAGGATTAGATACCCTGGTAGTCCATGCCGTAAACGTTGGGCGCTAGATGTAGGGACCTTT
CCACGGTTTCTGTGTCGTAGCTAACGCATTAAGCGCCCCGCCTGGGGAGTACGGCCGCAAGGCTAAAACTCAAAGGAATTGACGGGGGCCCGCACAAGCGGCGGAGCATGCGGATT
AATTCGATGCAACGCGAAGAACCTTACCAAGGCTTGACATACACCGGAAACGGCCAGAGATGGTCGCCCCCTTGTGGTCGGTGTACAGGTGGTGCATGGTTGTCGTCAGCTCGTGTC
GTGAGATGTTGGGTTAAGTCCCGCAACGAGCGCAACCCTCGTTCTATGTTGCCAGCGGGTTATGCCGGGGACTCATAGGAGACTGCCGGGGTCAACTCGGAGGAAGGTGGGGATGA
CGTCAAATCATCATGCCCCTTATGTCTTGGGCTTCACGCATGCTACAATGGCCGGTACAAAGGGCTGCGATACCGTAAGGTGGAGCGAATCCCAAAAAGCCGGTCTCAGTTCGGATTG
AGGTCTGCAACTCGACCTCATGAAGTCGGAGTCGCTAGTAATCGCAGATCAGCAACGCTGCGGTGAATACGTTCCCGGGCCTTGTACACACCGCCCGTCAAGTCATGAAAGTCGGTAA
CACCCGAAGCCGGTGGCCTAACCCTTGTGGAAGGAGCCGTCGAAGGTGGGATCGGTGATTAGGACTAAGTCGTAACAAGGTAGCCGTACCGGAAGGTGCGGCTGGATCACCTCCTT

Curtobacterium flaccumfaciens, Sequence ID AJ310414.1, 1514 bp., Percent identity 100%

AGAGTTTGATCCTGGCTCAGGACGAACGCTGGCGGCGTGCTTAACACATGCAAGTCGAACGATGATGCCCAGCTTGCTGGGTGGATTAGTGGCGAACGGGTGAGTAACACGTGAGT
AACCTGCCCCTGACTCTGGGATAAGCGTTGGAAACGACGTCTAATACTGGATATGATCACTGGCCGCATGGTCTGGTGGTGGAAAGATTTTTTGGTTGGGGATGGACTCGCGGCCTAT
CAGCTTGTTGGTGAGGTAATGGCTCACCAAGGCGACGACGGGTAGCCGGCCTGAGAGGGTGACCGGCCACACTGGGACTGAGACACGGCCCAGACTCCTACGGGAGGCAGCAGTG
GGGAATATTGCACAATGGGCGAAAGCCTGATGCAGCAACGCCGCGTGAGGGATGACGGCCTTCGGGTTGTAAACCTCTTTTAGTAGGGAAGAAGCGAAAGTGACGGTACCTGCAGA
AAAAGCACCGGCTAACTACGTGCCAGCAGCCGCGGTAATACGTAGGGTGCAAGCGTTGTCCGGAATTATTGGGCGTAAAGAGCTCGTAGGCGGTTTGTCGCGTCTGCTGTGAAATCC
CGAGGCTCAACCTCGGGCTTGCAGTGGGTACGGGCAGACTAGAGTGCGGTAGGGGAGATTGGAATTCCTGGTGTAGCGGTGGAATGCGCAGATATCAGGAGGAACACCGATGGCG
AAGGCAGATCTCTGGGCCGTAACTGACGCTGAGGAGCGAAAGCATGGGGAGCGAACAGGATTAGATACCCTGGTAGTCCATGCCGTAAACGTTGGGCGCTAGATGTAGGGACCTTT
CCACGGTTTCTGTGTCGTAGCTAACGCATTAAGCGCCCCGCCTGGGGAGTACGGCCGCAAGGCTAAAACTCAAAGGAATTGACGGGGGCCCGCACAAGCGGCGGAGCATGCGGATT
AATTCGATGCAACGCGAAGAACCTTACCAAGGCTTGACATACACCGGAAACGGCCAGAGATGGTCGCCCCCTTGTGGTCGGTGTACAGGTGGTGCATGGTTGTCGTCAGCTCGTGTC
GTGAGATGTTGGGTTAAGTCCCGCAACGAGCGCAACCCTCGTTCTATGTTGCCAGCGGGTTATGCCGGGGACTCATAGGAGACTGCCGGGGTCAACTCGGAGGAAGGTGGGGATGA
CGTCAAATCATCATGCCCCTTATGTCTTGGGCTTCACGCATGCTACAATGGCCGGTACAAAGGGCTGCGATACCGTAAGGTGGAGCGAATCCCAAAAAGCCGGTCTCAGTTCGGATTG
AGGTCTGCAACTCGACCTCATGAAGTCGGAGTCGCTAGTAATCGCAGATCAGCAACGCTGCGGTGAATACGTTCCCGGGCCTTGTACACACCGCCCGTCAAGTCATGAAAGTCGGTAA
CACCCGAAGCCGGTGGCCTAACCCTTGTGGAAGGAGCCGTCGAAGGTGGGATCGGTGATTAGGACTAAGTCGTAACAAGGTAGCCGTACCGGAAGGTGCGGCTGGATCACCTCCTT

4. ***Erwinia* sp.,** Sequence ID MZ424741, (s1860-4,10,16,22), 1508 bp.

AAGAGTTTGATCATGGCTCAGATTGAACGCTGGCGGCAGGCCTAACACATGCAAGTCGAACGGTAGCACAGAGAGCTTGCTCTCGGGTGACGAGTGGCGGACGGGTGAGTAATGTC
TGGGAAACTGCCTGATGGAGGGGGATAACTACTGGAAACGGTAGCTAATACCGCATAACGTCTTCGGACCAAAGTGGGGGACCTTCGGGCCTCACACCATCGGATGTGCCCAGATG
GGATTAGCTAGTAGGTGGGGTAATGGCTCACCTAGGCGACGATCCCTAGCTGGTCTGAGAGGATGACCAGCCACACTGGAACTGAGACACGGTCCAGACTCCTACGGGAGGCAGCA
GTGGGGAATATTGCACAATGGGCGCAAGCCTGATGCAGCCATGCCGCGTGTATGAAGAAGGCCTTCGGGTTGTAAAGTACTTTCAGCGGGGAGGAAGGCGATAAGGTTAATAACCT
TGTCGATTGACGTTACCCGCAGAAGAAGCACCGGCTAACTCCGTGCCAGCAGCCGCGGTAATACGGAGGGTGCAAGCGTTAATCGGAATTACTGGGCGTAAAGCGCACGCAGGCGG

TCTGTCAAGTCAGATGTGAAATCCCCGGGCTTAACCTGGGAACTGCATTTGAAACTGGCAGGCTAGAGTCTTGTAGAGGGGGGTAGAATTCCAGGTGTAGCGGTGAAATGCGTAGA
GATCTGGAGGAATACCGGTGGCGAAGGCGGCCCCCTGGACAAAGACTGACGCTCAGGTGCGAAAGCGTGGGGAGCAAACAGGATTAGATACCCTGGTAGTCCACGCCGTAAACGA
TGTCGACTTGGAGGTTGTGCCCTTGAGGCGTGGCTTCCGGAGCTAACGCGTTAAGTCGACCGCCTGGGGAGTACGGCCGCAAGGTTAAAACTCAAATGAATTGACGGGGGCCCGCA
CAAGCGGTGGAGCATGTGGTTTAATTCGATGCAACGCGAAGAACCTTACCTGGCCTTGACATCCACGGAATTCGGCAGAGATGCCTTAGTGCCTTCGGGAACCGTGAGACAGGTGCT
GCATGGCTGTCGTCAGCTCGTGTTGTGAAATGTTGGGTTAAGTCCCGCAACGAGCGCAACCCTTATCCTTTGTTGCCAGCGAGTAATGTCGGGAACTCAAAGGAGACTGCCGGTGACA
AACCGGAGGAAGGTGGGGATGACGTCAAGTCATCATGGCCCTTACGGCCAGGGCTACACACGTGCTACAATGGCGCATACAAAGAGAAGCGAACTCGCGAGAGCAAGCGGACCTC
ACAAAGTGCGTCGTAGTCCGGATCGGAGTCTGCAACTCGACTCCGTGAAGTCGGAATCGCTAGTAATCGTAGATCAGAATGCTACGGTGAATACGTTCCCGGGCCTTGTACACACCGC
CCGTCACACCATGGGAGTGGGTTGCAAAAGAAGTAGGTAGCTTAACCTTCGGGAGGGCGCTTACCACTTTGTGATTCATGACTGGGGTGAAGTCGTAACAAGGTAACCGTAGG

Erwinia billingiae, Sequence ID KM408608.1, 1535 bp., Percent identity 100%

GAAGAGTTTGATCATGGCTCAGATTGAACGCTGGCGGCAGGCCTAACACATGCAAGTCGAACGGTAGCACAGAGAGCTTGCTCTCGGGTGACGAGTGGCGGACGGGTGAGTAATGT
CTGGGAAACTGCCTGATGGAGGGGGATAACTACTGGAAACGGTAGCTAATACCGCATAACGTCTTCGGACCAAAGTGGGGGACCTTCGGGCCTCACACCATCGGATGTGCCCAGAT
GGGATTAGCTAGTAGGTGGGGTAATGGCTCACCTAGGCGACGATCCCTAGCTGGTCTGAGAGGATGACCAGCCACACTGGAACTGAGACACGGTCCAGACTCCTACGGGAGGCAGC
AGTGGGGAATATTGCACAATGGGCGCAAGCCTGATGCAGCCATGCCGCGTGTATGAAGAAGGCCTTCGGGTTGTAAAGTACTTTCAGCGGGGAGGAAGGCGATAAGGTTAATAACC
TTGTCGATTGACGTTACCCGCAGAAGAAGCACCGGCTAACTCCGTGCCAGCAGCCGCGGTAATACGGAGGGTGCAAGCGTTAATCGGAATTACTGGGCGTAAAGCGCACGCAGGCG
GTCTGTCAAGTCAGATGTGAAATCCCCGGGCTTAACCTGGGAACTGCATTTGAAACTGGCAGGCTAGAGTCTTGTAGAGGGGGGTAGAATTCCAGGTGTAGCGGTGAAATGCGTAG
AGATCTGGAGGAATACCGGTGGCGAAGGCGGCCCCCTGGACAAAGACTGACGCTCAGGTGCGAAAGCGTGGGGAGCAAACAGGATTAGATACCCTGGTAGTCCACGCCGTAAACG
ATGTCGACTTGGAGGTTGTGCCCTTGAGGCGTGGCTTCCGGAGCTAACGCGTTAAGTCGACCGCCTGGGGAGTACGGCCGCAAGGTTAAAACTCAAATGAATTGACGGGGGCCCGC
ACAAGCGGTGGAGCATGTGGTTTAATTCGATGCAACGCGAAGAACCTTACCTGGCCTTGACATCCACGGAATTCGGCAGAGATGCCTTAGTGCCTTCGGGAACCGTGAGACAGGTGC
TGCATGGCTGTCGTCAGCTCGTGTTGTGAAATGTTGGGTTAAGTCCCGCAACGAGCGCAACCCTTATCCTTTGTTGCCAGCGAGTAATGTCGGGAACTCAAAGGAGACTGCCGGTGAC
AAACCGGAGGAAGGTGGGGATGACGTCAAGTCATCATGGCCCTTACGGCCAGGGCTACACACGTGCTACAATGGCGCATACAAAGAGAAGCGAACTCGCGAGAGCAAGCGGACCT
CACAAAGTGCGTCGTAGTCCGGATCGGAGTCTGCAACTCGACTCCGTGAAGTCGGAATCGCTAGTAATCGTAGATCAGAATGCTACGGTGAATACGTTCCCGGGCCTTGTACACACCG
CCCGTCACACCATGGGAGTGGGTTGCAAAAGAAGTAGGTAGCTTAACCTTCGGGAGGGCGCTTACCACTTTGTGATTCATGACTGGGGTGAAGTCGTAACAAGGTAACCGTAGGGG
AACCTGCGGTTGGATCACCTCCTT

5. ***Gordonia aichiensis*** P6PL2. The complete genome has been sequenced and can be found in the following NCBI database “BioProject PRJNA1267753”. Ananev, A.A.; Aleynova, O.A.; Nityagovsky, N.N.; Suprun, A.R.; Ogneva, Z.V.; Kiselev, K.V. Whole Genome of *Gordonia aichiensis* P6PL2 Associated with *Vitis amurensis* That Stimulates Plant Growth. Horticulturae 2025, 11, 735. https://doi.org/10.3390/horticulturae11070735”.

6. *Pantoea* sp., Sequence ID MZ424742, (s1706-9-al14), 419 bp.

GCTGGCAGGCCTAACACATGCAAGTCGGACGGTAGCACAGAGGAGCTTGCTCCTTGGTGGACGAGTGCCGAACGGGTAAGTAATGTCGGGGAACCGCCCCGTAAAAGGGGGATAA
CCACTGGAAACGGTGGCTAATACCGCATAACGTCGCAAGACAAAAGAGGGGACCTTCGGGCCTCTCACTATCGGATGAAACCAGATGGGATTAGCTAGTAGGCGGGGTAATGGCCC

AACTAGGCGACCAACCCTAACCGGGCTGAAAGGAAGGACCAGCAACACGGAAATGAGAAAACGTCCAGAATCCCACCGGGAGGCACCAGTGGGGAAAATTGCACAATGGGCGCAA
GCCTGATGCAGCCATGCCGCGTGTATGAAGAAGGCCTTCGGGTTGTAAAGTACTTTCAGCGGGGAGGAAGGCG

***Pantoea agglomerans*,** Sequence ID MT605813.1, 1505bp., Percent identity 99.75%

AGAGTTTGATCATGGCTCAGATTGAACGCTGGCGGCAGGCCTAACACATGCAAGTCGGACGGTAGCACAGAGGAGCTTGCTCCTTGGGTGACGAGTGGCGGACGGGTGAGTAATGT
CTGGGGATCTGCCCGATAGAGGGGGATAACCACTGGAAACGGTGGCTAATACCGCATAACGTCGCAAGACCAAAGAGGGGGACCTTCGGGCCTCTCACTATCGGATGAACCCAGAT
GGGATTAGCTAGTAGGCGGGGTAATGGCCCACCTAGGCGACGATCCCTAGCTGGTCTGAGAGGATGACCAGCCACACTGGAACTGAGACACGGTCCAGACTCCTACGGGAGGCAGC
AGTGGGGAATATTGCACAATGGGCGCAAGCCTGATGCAGCCATGCCGCGTGTATGAAGAAGGCCTTCGGGTTGTAAAGTACTTTCAGCGGGGAGGAAGGCGACGGGGTTAATAACC
CTGTCGATTGACGTTACCCGCAGAAGAAGCACCGGCTAACTCCGTGCCAGCAGCCGCGGTAATACGGAGGGTGCAAGCGTTAATCGGAATTACTGGGCGTAAAGCGCACGCAGGCG
GTCTGTTAAGTCAGATGTGAAATCCCCGGGCTTAACCTGGGAACTGCATTTGAAACTGGCAGGCTTGAGTCTTGTAGAGGGGGGTAGAATTCCAGGTGTAGCGGTGAAATGCGTAGA
GATCTGGAGGAATACCGGTGGCGAAGGCGGCCCCCTGGACAAAGACTGACGCTCAGGTGCGAAAGCGTGGGGAGCAAACAGGATTAGATACCCTGGTAGTCCACGCCGTAAACGA
TGTCGACTTGGAGGTTGTTCCCTTGAGGAGTGGCTTCCGGAGCTAACGCGTTAAGTCGACCGCCTGGGGAGTACGGCCGCAAGGTTAAAACTCAAATGAATTGACGGGGGCCCGCAC
AAGCGGTGGAGCATGTGGTTTAATTCGATGCAACGCGAAGAACCTTACCTACTCTTGACATCCACGGAATTTGGCAGAGATGCCTTAGTGCCTTCGGGAACCGTGAGACAGGTGCTG
CATGGCTGTCGTCAGCTCGTGTTGTGAAATGTTGGGTTAAGTCCCGCAACGAGCGCAACCCTTATCCTTTGTTGCCAGCGATTCGGTCGGGAACTCAAAGGAGACTGCCGGTGATAAA
CCGGAGGAAGGTGGGGATGACGTCAAGTCATCATGGCCCTTACGAGTAGGGCTACACACGTGCTACAATGGCGCATACAAAGAGAAGCGACCTCGCGAGAGCAAGCGGACCTCACA
AAGTGCGTCGTAGTCCGGATCGGAGTCTGCAACTCGACTCCGTGAAGTCGGAATCGCTAGTAATCGTGGATCAGAATGCCACGGTGAATACGTTCCCGGGCCTTGTACACACCGCCC
GTCACACCATGGGAGTGGGTTGCAAAAGAAGTAGGTAGCTTAACCTTCGGGAGGGCGCTTACCACTTTGTGATTCATGACTGGGGTGAAGTCGTAACAAGGTACCATC

7. ***Pseudomonas* sp.,** Sequence ID MZ424743, (s1860-5,11,17,23), 1530 bp.

AAGAGTTTGATCATGGCTCAGATTGACCGCTGGCGGCAGGCCTAACACATGCAAGTCGAGCGGATGAGAGGAGCTTGCTCTTCGATTCAGCGGCGGACGGGTGAGTAATGCCTAGG
AATCTGCCTGGTAGTGGGGGACAACGTTTCGAAAGGAACGCTAATACCGCATACGTCCTACGGGAGAAAGCAGGGGACCTTCGGGCCTTGCGCTATCAGATGAGCCTAGGTCGGATT
AGCTAGTTGGTGAGGTAATGGCTCACCAAGGCGACGATCCGTAACTGGTCTGAGAGGATGATCAGTCACACTGGAACTGAGACACGGTCCAGACTCCTACGGGAGGCAGCAGTGGG
GAATATTGGACAATGGGCGAAAGCCTGATCCAGCCATGCCGCGTGTGTGAAGAAGGTCTTCGGATTGTAAAGCACTTTAAGTTGGGAGGAAGGGTTGTTTCCTAATACGAAGCAATT
TTGACGTTACCGACAGAATAAGCACCGGCTAACTCTGTGCCAGCAGCCGCGGTAATACAGAGGGTGCAAGCGTTAATCGGAATTACTGGGCGTAAAGCGCGCGTAGGTGGTTTGTTA
AGTTGAATGTGAAATCCCCGGGCTCAACCTGGGAACTGCATCCAAAACTGGCAAGCTAGAGTAGGGCAGAGGGTGGTGGAATTTCCTGTGTAGCGGTGAAATGCGTAGATATAGGA
AGGAACACCAGTGGCGAAGGCGACCACCTGGGCTCATACTGACACTGAGGTGCGAAAGCGTGGGGAGCAAACAGGATTAGATACCCTGGTAGTCCACGCCGTAAACGATGTCAACT
AGCCGTTGGAATCCTTGAGATTTTAGTGGCGCAGCTAACGCATTAAGTTGACCGCCTGGGGAGTACGGCCGCAAGGTTAAAACTCAAATGAATTGACGGGGGCCCGCACAAGCGGT
GGAGCATGTGGTTTAATTCGAAGCAACGCGAAGAACCTTACCAGGCCTTGACATCCAATGAACTTTCCAGAGATGGATTGGTGCCTTCGGGAACATTGAGACAGGTGCTGCATGGCT
GTCGTCAGCTCGTGTCGTGAGATGTTGGGTTAAGTCCCGTAACGAGCGCAACCCTTGTCCTTAGTTACCAGCACGTTATGGTGGGCACTCTAAGGAGACTGCCGGTGACAAACCGGA
GGAAGGTGGGGATGACGTCAAGTCATCATGGCCCTTACGGCCTGGGCTACACACGTGCTACAATGGTCGGTACAGAGGGTTGCCAAGCCGCGAGGTGGAGCTAATCTCACAAAACC
GATCGTAGTCCGGATCGCAGTCTGCAACTCGACTGCGTGAAGTCGGAATCGCTAGTAATCGCGAATCAGAATGTCGCGGTGAATACGTTCCCGGGCCTTGTACACACCGCCCGTCACA

CCATGGGAGTGGGTTGCACCAGAAGTAGCTAGTCTAACCTTCGGGAGGACGGTTACCACGGTGTGATTCATGACTGGGGTGAAGTCGTAACAAGGTAGCCGTAGGGGAACCTGCGG
CTGGATCACCTCCTT

Pseudomonas alkylphenolica, Sequence ID MN813762.1, 1542 bp., Percent identity 98.89%

ATTGAACTGAAGAGTTTGATCATGGCTCAGATTGAACGCTGGCGGCAGGCCTAACACATGCAAGTCGAGCGGATGAGAAGAGCTTGCTCTTCGATTCAGCGGCGGACGGGTGAGTA
ATACCTAGGAATCTGCCTGGTAGTGGGGGACAACGTTTCGAAAGGAACGCTAATACCGCATACGTCCTACGGGAGAAAGCAGGGGACCTTCGGGCCTTGCGCTATCAGATGAGCCTA
GGTCGGATTAGCTAGTTGGTGAGGTAATGGCTCACCAAGGCGACGATCCGTAACTGGTCTGAGAGGATGATCAGTCACACTGGAACTGAGACACGGTCCAGACTCCTACGGGAGGC
AGCAGTGGGGAATATTGGACAATGGGCGAAAGCCTGATCCAGCCATGCCGCGTGTGTGAAGAAGGTCTTCGGATTGTAAAGCACTTTAAGTTGGGAGGAAGGGTACTTACCTAATA
CGTGAGTATTTTGACGTTACCGACAGAATAAGCACCGGCTAACTCTGTGCCAGCAGCCGCGGTAATACAGAGGGTGCAAGCGTTAATCGGAATTACTGGGCGTAAAGCGCGCGTAGG
TGGTTTGTTAAGTTGGATGTGAAATCCCCGGGCTCAACCTGGGAACTGCATCCAAAACTGGCAAGCTAGAGTAGGGCAGAGGGTGGTGGAATTTCCTGTGTAGCGGTGAAATGCGTA
GATATAGGAAGGAACACCAGTGGCGAAGGCGACCACCTGGGCTCATACTGACACTGAGGTGCGAAAGCGTGGGGAGCAAACAGGATTAGATACCCTGGTAGTCCACGCCGTAAAC
GATGTCAACTAGCCGTTGGAATCCTTGAGATTTTAGTGGCGCAGCTAACGCATTAAGTTGACCGCCTGGGGAGTACGGCCGCAAGGTTAAAACTCAAATGAATTGACGGGGGCCCGC
ACAAGCGGTGGAGCATGTGGTTTAATTCGAAGCAACGCGAAGAACCTTACCAGGCCTTGACATGCAGAGAACTTTCCAGAGATGGATTGGTGCCTTCGGGAACTCTGACACAGGTGC
TGCATGGCTGTCGTCAGCTCGTGTCGTGAGATGTTGGGTTAAGTCCCGTAACGAGCGCAACCCTTGTCCTTAGTTACCAGCACGTTATGGTGGGCACTCTAAGGAGACTGCCGGTGAC
AAACCGGAGGAAGGTGGGGATGACGTCAAGTCATCATGGCCCTTACGGCCTGGGCTACACACGTGCTACAATGGTCGGTACAGAGGGTTGCCAAGCCGCGAGGTGGAGCTAATCTC
ACAAAACCGATCGTAGTCCGGATCGCAGTCTGCAACTCGACTGCGTGAAGTCGGAATCGCTAGTAATCGCGAATCAGAATGTCGCGGTGAATACGTTCCCGGGCCTTGTACACACCGC
CCGTCACACCATGGGAGTGGGTTGCACCAGAAGTAGCTAGTCTAACCTTCGGGAGGACGGTTACCACGGTGTGATTCATGACTGGGGTGAAGTCGTAACAAGGTAGCCGTAGGGGA
ACCTGCGGCTGGATCACCTCCTTAAT

8. ***Sphingomonas* sp.**, Sequence ID PX909750, (s2342-21,22,23,24), 1482 bp.

ATAAGGAGGTGATCCAGCCGCAGGTTCCCCTACGGCTACCTTGTTACGACTTCACCCCAGTCGCTAAACCCACCGTGGTCGCCTGCCTCTCATTGCTGAGTTAGCGCAACGCCTTCGGG
TGAATCCAACTCCCATGGTGTGACGGGCGGTGTGTACAAGGCCTGGGAACGTATTCACCGCGGCATGCTGATCCGCGATTACTAGCGATTCCGCCTTCATGCTCTCGAGTTGCAGAGA
ACAATCCGAACTGAGACGGCTTTTGGAGATTAGCTCACACTCGCGTGCTTGCTGCCCACTGTCACCGCCATTGTAGCACGTGTGTAGCCCAGCGCGTAAGGGCCATGAGGACTTGACG
TCATCCCCACCTTCCTCCGGCTTATCACCGGCGGTTACCTTAAAGTGCCCAACTAAATGATGGCAACTAAGGTCGAGGGTTGCGCTCGTTGCGGGACTTAACCCAACATCTCACGACAC
GAGCTGACGACAGCCATGCAGCACCTGTGTGCAGGTCCCCGAAGGGAAGAAATCCATCTCTGGAAGTCGTCCTGCCATGTCAAACGCTGGTAAGGTTCTGCGCGTTGCTTCGAATTAA
ACCACATGCTCCACCGCTTGTGCAGGCCCCCGTCAATTCATTTGAGTTTTAACCTTGCGGCCGTACTCCCCAGGCGGATAACTTAATGCGTTAGCTGCGCCACCCAAAGACCAAGTCCC
CGGACAGCTAGTTATCATCGTTTACGGCGTGGACTACCAGGGTATCTAATCCTGTTTGCTCCCCACGCTTTCGCACCTCAGCGTCAATACATGTCCAGTCAGCCGCCTTCGCCACTGGTG
TTCTTCCGAATATCTACGAATTTCACCTCTACACTCGGAATTCCACTGACCTCTCCATGATTCAAGCGATGCAGTCTAAAAGGCAATTCCAGAGTTGAGCTCTGGGCTTTCACCTCTTACT
TACAAAGCCGCCTACGTGCGCTTTACGCCCAGTAATTCCGAATAACGCTAGCTCCCTCCGTATTACCGCGGCTGCTGGCACGGAGTTAGCCGGAGCTTATTCTCCCGGTACTGTCATTA
TCATCCCGGGTAAAAGAGCTTTACAACCCTAAGGCCTTCATCACTCACGCGGCATTGCTGGATCAGGCTTTCGCCCATTGTCCAATATTCCCCACTGCTGCCTCCCGTAGGAGTCTGGG
CCGTGTCTCAGTCCCAGTGTGGCTGATCATCCTCTCAGACCAGCTAAGGATCGTCGGCTTGGTGCGCCTTTACCACACCAACTACCTAATCCTACGCGGGCTCATCCCTCGGCGATAAA

TCTTTGGACTTACGTCATCATCCGGTATTAGCAGTCGTTTCCAACTGTTATTCCGAACCAAGGGGCAGATTCCCACGCGTTACGCACCCGTGCGCCACTAAACCCGAAGGCTTCGTTCG
ACTTGCATGTGTTAGGCATGCCGCCAGCGTTCATTCTGAGCCATGATCAAACTC

Sphingomonas aerolata, Sequence ID CP098762, 1487 bp., Percent identity 99%

AAAGGAGGTGATCCAGCCGCAGGTTCCCCTACGGCTACCTTGTTACGACTTCACCCCAGTCGCTAAACCCACCGTGGTCGCCTGCCTCTCTTGCGAGTTAGCGCAACGCCTTCGGGTGA
ATCCAACTCCCATGGTGTGACGGGCGGTGTGTACAAGGCCTGGGAACGTATTCACCGCGGCATGCTGATCCGCGATTACTAGCGATTCCGCCTTCATGCTCTCGAGTTGCAGAGAACA
ATCCGAACTGAGACGGCTTTTGGAGATTAGCTCACACTCGCGTGCTTGCTGCCCACTGTCACCGCCATTGTAGCACGTGTGTAGCCCAGCGCGTAAGGGCCATGAGGACTTGACGTCA
TCCCCACCTTCCTCCGGCTTATCACCGGCGGTTACCTTAAAGTGCCCAACTAAATGATGGCAACTAAGGTCGAGGGTTGCGCTCGTTGCGGGACTTAACCCAACATCTCACGACACGAG
CTGACGACAGCCATGCAGCACCTGTGTGCAGGTCCCCGAAGGGAAGAAATCCATCTCTGGAAGTCGTCCTGCCATGTCAAACGCTGGTAAGGTTCTGCGCGTTGCTTCGAATTAAACC
ACATGCTCCACCGCTTGTGCAGGCCCCCGTCAATTCATTTGAGTTTTAACCTTGCGGCCGTACTCCCCAGGCGGATAACTTAATGCGTTAGCTGCGCCACCCAAAGACCAAGTCCCCGG
ACAGCTAGTTATCATCGTTTACGGCGTGGACTACCAGGGTATCTAATCCTGTTTGCTCCCCACGCTTTCGCACCTCAGCGTCAATACATGTCCAGTCAGCCGCCTTCGCCACTGGTGTTC
TTCCGAATATCTACGAATTTCACCTCTACACTCGGAATTCCACTGACCTCTCCATGATTCAAGCGATGCAGTCTAAAAGGCAATTCCAGAGTTGAGCTCTGGGCTTTCACCTCTTACTTAC
AAAGCCGCCTACGTGCGCTTTACGCCCAGTAATTCCGAATAACGCTAGCTCCCTCCGTATTACCGCGGCTGCTGGCACGGAGTTAGCCGGAGCTTATTCTCCCGGTACTGTCATTATCA
TCCCGGGTAAAAGAGCTTTACAACCCTAAGGCCTTCATCACTCACGCGGCATTGCTGGATCAGGCTTTCGCCCATTGTCCAATATTCCCCACTGCTGCCTCCCGTAGGAGTCTGGGCCG
TGTCTCAGTCCCAGTGTGGCTGATCATCCTCTCAGACCAGCTAAGGATCGTCGGCTTGGTGCGCCTTTACCACACCAACTACCTAATCCTACGCGGGCTCATCCCTCGGCGATAAATCTT
TGGACTTACGTCATCATCCGGTATTAGCAGTCGTTTCCAACTGTTATTCCGAACCAAGGGGCAGATTCCCACGCGTTACGCACCCGTGCGCCACTAAACCCGAAGGCTTCGTTCGACTT
GCATGTGTTAGGCATGCCGCCAGCGTTCATTCTGAGCCATGATCAAACTCTCAAGTTT

9. ***Xanthomonas* sp.**, Sequence ID MZ424744, (s1860-6,12,18,24), 1497 bp.

AGTGAACGCTGGCGGCAGGCCTAACACATGCAAGTCGAACGGCAGCACAGAGGAGCTTGCTCCTTGGGTGGCGAGTGGCGGACGGGTGAGGAATACATCGGAATCTACTCTGTCGT
GGGGGATAACGTAGGGAAACTTACGCTAATACCGCATACGACCTACGGGTGAAAGCGGAGGACCTTCGGGCTTCGCGCGATTGAATGAGCCGATGTCGGATTAGCTAGTTGGCGGG
GTAAAGGCCCACCAAGGCGACGATCCGTAGCTGGTCTGAGAGGATGATCAGCCACACTGGAACTGAGACACGGTCCAGACTCCTACGGGAGGCAGCAGTGGGGAATATTGGACAAT
GGGCGCAAGCCTGATCCAGCCATGCCGCGTGGGTGAAGAAGGCCTTCGGGTTGTAAAGCCCTTTTGTTGGGAAAGAAAAGCAGTCGGTTAATACCCGATTGTTCTGACGGTACCCAA
AGAATAAGCACCGGCTAACTTCGTGCCAGCAGCCGCGGTAATACGAAGGGTGCAAGCGTTACTCGGAATTACTGGGCGTAAAGCGTGCGTAGGTGGTGGTTTAAGTCCGTTGTGAA
AGCCCTGGGCTCAACCTGGGAATTGCAGTGGATACTGGGTCACTAGAGTGTGGTAGAGGGTAGCGGAATTCCCGGTGTAGCAGTGAAATGCGTAGAGATCGGGAGGAACATCTGTG
GCGAAGGCGGCTACCTGGACCAACACTGACACTGAGGCACGAAAGCGTGGGGAGCAAACAGGATTAGATACCCTGGTAGTCCACGCCCTAAACGATGCGAACTGGATGTTGGGTGC
AATTTGGCACGCAGTATCGAAGCTAACGCGTTAAGTTCGCCGCCTGGGGAGTACGGTCGCAAGACTGAAACTCAAAGGAATTGACGGGGGCCCGCACAAGCGGTGGAGTATGTGGT
TTAATTCGATGCAACGCGAAGAACCTTACCTGGTCTTGACATCCACGGAACTTTCCAGAGATGGATTGGTGCCTTCGGGAACCGTGAGACAGGTGCTGCATGGCTGTCGTCAGCTCGT
GTCGTGAGATGTTGGGTTAAGTCCCGCAACGAGCGCAACCCTTGTCCTTAGTTGCCAGCACGTAATGGTGGGAACTCTAAGGAGACCGCCGGTGACAAACCGGAGGAAGGTGGGGA
TGACGTCAAGTCATCATGGCCCTTACGACCAGGGCTACACACGTACTACAATGGTTAGGACAGAGGGCTGCAATCCCGCGAGGGTGAGCCAATCCCAGAAACCTAATCTCAGTCCGG
ATTGGAGTCTGCAACTCGACTCCATGAAGTCGGAATCGCTAGTAATCGCAGATCAGCATTGCTGCGGTGAATACGTTCCCGGGCCTTGTACACACCGCCCGTCACACCATGGGAGTTT
GTTGCACCAGAAGCAGGTAGCTTAACCTTCGGGAGGGCGCTTGCCACGGTGTGGCCGATGACTGGGGTGAAGTCGTAACAAGGTAGCCGTATCGGAAGG

Xanthomonas campestris, Sequence ID MN108237,1547 bp., Percent identity 99.13%

TAAGTGAAGAGTTTGATCCTGGCTCAGAGTGAACGCTGGCGGCAGGCCTAACACATGCAAGTCGAACGGCAGCACAGTAAGAGCTTGCTCTTATGGGTGGCGAGTGGCGGACGGGT
GAGGAATACATCGGAATCTACTCTTTCGTGGGGGATAACGTAGGGAAACTTACGCTAATACCGCATACGACCTACGGGTGAAAGCGGAGGACCTTCGGGCTTCGCGCGATTGAATGA
GCCGATGTCGGATTAGCTAGTTGGCGGGGTAAAGGCCCACCAAGGCGACGATCCGTAGCTGGTCTGAGAGGATGATCAGCCACACTGGAACTGAGACACGGTCCAGACTCCTACGG
GAGGCAGCAGTGGGGAATATTGGACAATGGGCGCAAGCCTGATCCAGCCATGCCGCGTGGGTGAAGAAGGCCTTCGGGTTGTAAAGCCCTTTTGTTGGGAAAGAAAAGCAGTCGGT
TAATACCCGATTGTTCTGACGGTACCCAAAGAATAAGCACCGGCTAACTTCGTGCCAGCAGCCGCGGTAATACGAAGGGTGCAAGCGTTACTCGGAATTACTGGGCGTAAAGCGTGC
GTAGGTGGTGGTTTAAGTCTGTTGTGAAAGCCCTGGGCTCAACCTGGGAATTGCAGTGGATACTGGGTCACTAGAGTGTGGTAGAGGGTAGCGGAATTCCCGGTGTAGCAGTGAAA
TGCGTAGAGATCGGGAGGAACATCCGTGGCGAAGGCGGCTACCTGGACCAACACTGACACTGAGGCACGAAAGCGTGGGGAGCAAACAGGATTAGATACCCTGGTAGTCCACGCC
CTAAACGATGCGAACTGGATGTTGGGTGCAATTTGGCACGCAGTATCGAAGCTAACGCGTTAAGTTCGCCGCCTGGGGAGTACGGTCGCAAGACTGAAACTCAAAGGAATTGACGG
GGGCCCGCACAAGCGGTGGAGTATGTGGTTTAATTCGATGCAACGCGAAGAACCTTACCTGGTCTTGACATCCACGGAACTTTCCAGAGATGGATTGGTGCCTTCGGGAACCGTGAG
ACAGGTGCTGCATGGCTGTCGTCAGCTCGTGTCGTGAGATGTTGGGTTAAGTCCCGCAACGAGCGCAACCCTTGTCCTTAGTTGCCAGCACGTAATGGTGGGAACTCTAAGGAGACC
GCCGGTGACAAACCGGAGGAAGGTGGGGATGACGTCAAGTCATCATGGCCCTTACGACCAGGGCTACACACGTACTACAATGGTAGGGACAGAGGGCTGCAAACCCGCGAGGGTA
AGCCAATCCCAGAAACCCTATCTCAGTCCGGATTGGAGTCTGCAACTCGACTCCATGAAGTCGGAATCGCTAGTAATCGCAGATCAGCATTGCTGCGGTGAATACGTTCCCGGGCCTT
GTACACACCGCCCGTCACACCATGGGAGTTTGTTGCACCAGAAGCAGGTAGCTTAACCTTCGGGAGGGCGCTTGCCACGGTGTGGCCGATGACTGGGGTGAAGTCGTAACAAGGTA
GCCGTATCGGAAGGTGCGGCTGGATCACCTCCTTT

10. ***Biscogniauxia* sp.**, Sequence ID MZ427923, (s1778-10-al22), 590 bp

ATGTGAACATACCTACTGTTGCCTCGGCAGGTCGTGCTGCGCGGCAGGATCGCCCCCCCTCGGGTTCGGCTGCTACAGGACTAGCTACCCTGTAGCGGCTTTCCTGGAGTGGCTACCC
TGGAGCAGCTGCTACTGGACTAGCTACCCCGTAGCAGCTGCCCTGGGGCGGCTACCCCGCAGCGGGGGGGCGCCCGCCCCGCAGCACGCCAACAGGCCTGCCGGAGGACCCCTAAA
CTCTGTTTTACACCTGTATCTCTGAGGCTATGATGGAAATAAGTTAAAACTTTCAACAACGGATCTCTTGGCTCTGGCATCGATGAAGAACGCAGCGAAATGCGATAAGTAATGTGAAT
TGCAGAATTCAGTGAATCATCGAATCTTTGAACGCACATTGCGCCTGACAGTATTCTGTTAGGCAAGCCTGTTCGAGCGTCATTTCAACCCTCAAGCCCTATTTGCTTGACGTTGGGAG
TTTACGGAGACGTAATTCCTTAAATATAGTGGCGGAGCCGGGTCGTGCTCTGGGCGTAGTAACCAAACTCTCGCCTCTGTAGCCGGCTCGGGTCTTGCCGTAAAACCCCCTATATTCT

Biscogniauxia maritima, Sequence ID MN341558.1, 707 bp., Percent identity 100%

GTAACAAGGTCTCCGTTGGTGAACCAGCGGAGGGATCATTAGCGAGTTGGTTACAAGCTCCAAACCCATGTGAACATACCTACTGTTGCCTCGGCAGGTCGTGCTGCGCGGCAGGAT
CGCCCCCCCTCGGGTTCGGCTGCTACAGGACTAGCTACCCTGTAGCGGCTACCCTGGAGTGGCTACCCTGGAGCAGCTGCTACTGGACTAGCTACCCCGTAGCAGCTGCCCTGGGGCA
GCTACCCCGCAGCGGGGGGGCGCCCGCCCCGCAGCACGCCAACAGGCCTGCCGGAGGACCCCTAAACTCTGTTTTACACCTGTATCTCTGAGGCTATGATGGAAATAAGTTAAAACTT
TCAACAACGGATCTCTTGGCTCTGGCATCGATGAAGAACGCAGCGAAATGCGATAAGTAATGTGAATTGCAGAATTCAGTGAATCATCGAATCTTTGAACGCACATTGCGCCTGACAG
TATTCTGTTAGGCATGCCTGTTCGAGCGTCATTTCAACCCTCAAGCCCTATTTGCTTGACGTTGGGAGTTTACGGAGACGTAATTCCTTAAATATAGTGGCGGAGCCGGGTCGTGCTCT
GGGCGTAGTAACCAAACTCTCGCCTCTGTAGCCGGCTCGGGTCTTGCCGTAAAACCCCCTATATTCTTCTGGTTGACCTCGGATCAGGTAGGAATACCCGCTGAACTTAAGCATATC

11. ***Cladosporium* sp.**, Sequence ID MZ427924, (s1778-7-al19), 512 bp.

AGGGATCATTACAAGTGACCCCGGTCTTACCACCGGGATGTTCATAACCCTTTGTTGTCCGACTCTGTTGCCTCCGGGGCGACCCTGCCTTCGGGCGGGGGCTCCGGGTGGACACTTC
AAACTCTTGCGTAACTTTGCAGTCTGAGTAAACTTAATTAATAAATTAAAACTTTTAACAACGGATCTCTTGGTTCTGGCATCGATGAAGAACGCAGCGAAATGCGATAAGTAATGTGA
ATTGCAGAATTCAGTGAATCATCGAATCTTTGAACGCACATTGCGCCCCCTGGTATTCCGGGGGGCATGCCTGTTCGAGCGTCATTTCACCACTCAAGCCTCGCTTGGTATTGGGCAAC
GCGGTCCGCCGCGTGCCTCAAATCGACCGGCTGGGTCTTCTGTCCCCTAAGCGTTGTGGAAACTATTCGCTAAAGGGTGTTCGGGAGGCTACGCCGTAAAACAACCCCATTTCTAAGG
TTGACCTCGGATCAGGTAGGGATACCCGCTGAACTTAA

Cladosporium perangustum, Sequence ID MT645918.1, 512 bp., Percent identity 100%

AGGGATCATTACAAGTGACCCCGGTCTTACCACCGGGATGTTCATAACCCTTTGTTGTCCGACTCTGTTGCCTCCGGGGCGACCCTGCCTTCGGGCGGGGGCTCCGGGTGGACACTTC
AAACTCTTGCGTAACTTTGCAGTCTGAGTAAACTTAATTAATAAATTAAAACTTTTAACAACGGATCTCTTGGTTCTGGCATCGATGAAGAACGCAGCGAAATGCGATAAGTAATGTGA
ATTGCAGAATTCAGTGAATCATCGAATCTTTGAACGCACATTGCGCCCCCTGGTATTCCGGGGGGCATGCCTGTTCGAGCGTCATTTCACCACTCAAGCCTCGCTTGGTATTGGGCAAC
GCGGTCCGCCGCGTGCCTCAAATCGACCGGCTGGGTCTTCTGTCCCCTAAGCGTTGTGGAAACTATTCGCTAAAGGGTGTTCGGGAGGCTACGCCGTAAAACAACCCCATTTCTAAGG
TTGACCTCGGATCAGGTAGGGATACCCGCTGAACTTAA

12. ***Didymella* sp.,** Sequence ID MZ427926, (s1778-5-al17), 543 bp.

AGTCGTAACAAGGTTTCCGTAGGTGAACCTGCGGAAGGATCATTACCTAGAGTTGTGGGCTTTGCCCGCCATCTCTTACCCATGTCTTTTGAGTACCTTCGTTTCCTCGGCGGGTCCGC
CCGCCGATTGGACAATTTAAACCATTTGCAGTTGCAATCAGCGTCTGAAAAAAACTTAATAGTTACAACTTTCAACAACGGATCTCTTGGTTCTGGCATCGATGAAGAACGCAGCGAAA
TGCGATAAGTAGTGTGAATTGCAGAATTCAGTGAATCATCGAATCTTTGAACGCACATTGCGCCCCTTGGTATTCCATGGGGCATGCCTGTTCGAGCGTCATTTGTACCTTCAAGCTCT
GCTTGGTGTTGGGTGTTTGTCTCGCCTCCGCGTGTAGACTCGCCTCAAAACAATTGGCAGCCGGCGTATTGATTTCGGAGCGCAGTACATCTCGCGCTTTGCACTCATAACGACGACGT
CCAAAAGTACATTTTTTACACTCTTGACCTCGGATCAGGTAGGGATACCCGCTGAACTTAAGCATAT

Didymella pinodella, Sequence ID KX869956.1, 957 bp., Percent identity 100%

TCTTGGTCCATTTAGAGGAAGTAAAAGTCGTAACAAGGTTTCCGTAGGTGAACCTGCGGAAGGATCATTACCTAGAGTTGTGGGCTTTGCCCGCCATCTCTTACCCATGTCTTTTGAGT
ACCTTCGTTTCCTCGGCGGGTCCGCCCGCCGATTGGACAATTTAAACCATTTGCAGTTGCAATCAGCGTCTGAAAAAAACTTAATAGTTACAACTTTCAACAACGGATCTCTTGGTTCTG
GCATCGATGAAGAACGCAGCGAAATGCGATAAGTAGTGTGAATTGCAGAATTCAGTGAATCATCGAATCTTTGAACGCACATTGCGCCCCTTGGTATTCCATGGGGCATGCCTGTTCG
AGCGTCATTTGTACCTTCAAGCTCTGCTTGGTGTTGGGTGTTTGTCTCGCCTCCGCGTGTAGACTCGCCTCAAAACAATTGGCAGCCGGCGTATTGATTTCGGAGCGCAGTACATCTCG
CGCTTTGCACTCATAACGACGACGTCCAAAAGTACATTTTTTACACTCTTGACCTCGGATCAGGTAGGGATACCCGCTGAACTTAAGCATATCAATAAGCGGAGGAAAAGAAACCAAC
AGGGATTGCCCTAGTAACGGCGAGTGAAGCGGCAACAGCTCAAATTTGAAATCTGGCGTCTTTGGCGTCCGAGTTGTAATTTGCAGAGGGCGCTTTGGCATTGGCAGCGGTCCAAGT
TCCTTGGAACAGGACGTCACAGAGGGTGAGAATCCCGTACGTGGTCGCTAGCCTTTACCGTGTAAAGCCCCTTCGACGAGTCGAGTTGTTTGGGAATGCAGCTCTAAATGGGAGGTA
AATTTCTTCTAAAGCTAAATACTGGCCAGAGACCGATAGCGCACAAGTAGAGTGATCGAAAGATGAAAAGCACTTTGGAAAGAGAGTTAAAAAGCACGTGAAATTGTTGAAAGGAA
AAAACACTTGAAA

13. ***Exobasidium* sp.**, Sequence ID PX916210, (s2341-3-4), 396 bp.

AACCCCTGTGATCGGTGCTGAGTTCGCTCATGGCACCCCATTTTATCACACTTTATAGTTTGAACGTAAAAACAAAAAACAAAATATACTTTTGACAACGGATCTCTGGTTCTCCCATCG
ATGAAGAACGCAGCGAAATGCGATAAGTAATGTGAATTGCAGAATTCAGTGAATCATCGAATCTTTGAACGCACCTTGCGCTCCTTGGTATTCCTTGGAGCATGCCTGTTTGAGTGTCT
TGAATATCTCTGCTCCCCAGTTTTTTAATTAAGGCTGGTTTGGAGTCAGGTCCTTGGGCTTGCTTGCGGTTTGTTACGAACACAAGCTTGCCTTAAATGTATTAGCTGGATTTCAGTAGA
GTTGTTAAGTAACATTGAAAACTCTCGTAAACGAAGT

Exobasidium japonicum, Sequence ID EU692773.1, 592 bp., Percent identity 96%

TTTCTGTAGGTGAACCTGCAGAAGGATCATTATTGAATTGTAAAAATGGGGAGTTGCTTTCGAGCACTCCTCTATATTCTTAATTCCTAAACACCTGTGAATCGTTGCTGAGCTCGCTCA
GTGGCACAATCTTTTATACACACTTTATAGTTTTGAACGTAACAAAAAACAAAAAACAAAAATATAACTTTTGACAACGGATCTCTTGGTTCTCCCATCGATGAAGAACGCAGCGAAAT
GCGATAAGTAATGTGAATTGCAGAATTCAGTGAATCATCGAATCTTTGAACGCACCTTGCGCTCCTTGGTATTCCTTGGAGCATGCCTGTTTGAGTGTCTTGAATATCTCTGCTCCTCAA
TTTTTTAATTAAAGTTGGTTTGGAGTCAGGTCCTTGGGCTTGCTTGCGGTTTGTTACGAACACAAGCTTGCCTTAAATGTATTAGCTGGATTTCAGTAGAGTTGTTAAGTAACATTGAAA
ACTCTCGTAATACGAAGTCTGCTTTCTAACCCGTCGCTTCTTTTTTCACGAAAAGTCGCACACATATTCATTTCTTTGGCCTCAAATCAGGTAGGACTACCCGCTGAACTTAA

14. ***Penicillium* sp.**, Sequence ID PX916211, (s2344-3-4), 278 bp.

GGAAGTAAAAAGTCGTAACAAGGTTTCCGTAGGTGAACCTGCGGAAGGATCATTACCGAGTGAGGGCCCTCTGGGTCCAACCTCCCACCCGTGTTTATTTTACCTTGTTGCTTCGGCG
AGCCTGCCTTTTGGCTGCCGGGGGACGTCTGTCCCCGGGTCCGCGCTCGCCGAAGACACCTTAGAACTCTGTCTGAAGATTGTAGTCTGAGATTAAATATAAATTATTTAAAACTTTCA
ACAACGGATCTCTTGGTTCCGGCATCGATGATGAACGCAGC

Penicillium brevicompactum, Sequence ID MW018697.1, 534 bp., Percent identity 96%

TGGAAGTAAAAAGTCGTAACAAGGTTTCCGTAGGTGAACCTGCGGAAGGATCATTACCGAGTGAGGGCCCTCTGGGTCCAACCTCCCACCCGTGTTTATTTTACCTTGTTGCTTCGGC
GAGCCTGCCTTTTGGCTGCCGGGGGACGTCTGTCCCCGGGTCCGCGCTCGCCGAAGACACCTTAGAACTCTGTCTGAAGATTGTAGTCTGAGATTAAATATAAATTATTTAAAACTTTC
AACAACGGATCTCTTGGTTCCGGCATCGATGAAGAACGCAGCGAAATGCGATACGTAATGTGAATTGCAGAATTCAGTGAATCATCGAGTCTTTGAACGCACATTGCGCCCTCTGGTA
TTCCGGAGGGCATGCCTGTCCGAGCGTCATTGCTGCCCTCAAGCACGGCTTGTGTGTTGGGCTCCGTCCTCCTTCCGGGGGACGGGCCCGAAAGGCAGCGGCGGCACCGCGTCCGGT
CCTCAAGCGTATGGGGCTTTGTCACCCGCTTTGTAGGACTGGCCGGCGCCTGCCGATCAACC

15. ***Pestalotiopsis* sp.**, Sequence ID PX916212, (s2341-9-10), 563 bp.

ATATAGGAGAAGTCGTAACAAGGTCTCCGTTGGTGAACCAGCGGAGGGATCATTATAGAGTTTTCTAAACTCCCAACCCATGTGAACTTACCATTGTTGCCTCGGCAGAAGCTGCTCG
GTGCACCCTACCTTGGAACGGCCTACCCTGTAGCGCCTTACCCTGGAACGGCTTACCCTGTAGCGGCTGCCGGTGGACTACCAAACTCTTGTTATTTTATTGTAATCTGAGCGTCTTATT
TTAATAAGTCAAAACTTTCAACAACGGATCTCTTGGTTCTGGCATCGATGAAGAACGCAGCGAAATGCGATAAGTAATGTGAATTGCAGAATTCAGTGAATCATCGAATCTTTGAACG

CACATTGCGCCCATTAGTATTCTAGTGGGCATGCCTGTTCGAGCGTCATTTCAACCCTTAAGCCTAGCTTAGTGTTGGGAGCCTACTGCTTTTACTAGCTGTAGCTCCTGAAATACAACG
GCGGATCTGCGATATCCTCTGAGCGTAGTAATTTTTATCTCGCTTTTGACTGGAGTTGCAGCGTCTTTAGCCGCTAAATCCCCCAAT

Pestalotiopsis biciliate, Sequence ID PP146582.1, 706 bp., Percent identity 99%

CACACCGCCCGTCGCTACTACCGATTGAATGGCTCAGTGAGGCTTTCGGACTGGCCCAGGGAGGTCGGCAACGACCACCCAGGGCCGGAAAGTTATCCAAACTCGGTCATTTAGAGG
AAGTAAAAGTCGTAACAAGGTCTCCGTTGGTGAACCAGCGGAGGGATCATTATAGAGTTTTCTAAACTCCCAACCCATGTGAACTTACCATTGTTGCCTCGGCAGAAGCTGCTCGGTG
CACCCTACCTTGGAACGGCCTACCCTGTAGCGCCTTACCCTGGAACGGCTTACCCTGTAGCGGCTGCCGGTGGACTACCAAACTCTTGTTATTTTATTGTAATCTGAGCGTCTTATTTTA
ATAAGTCAAAACTTTCAACAACGGATCTCTTGGTTCTGGCATCGATGAAGAACGCAGCGAAATGCGATAAGTAATGTGAATTGCAGAATTCAGTGAATCATCGAATCTTTGAACGCAC
ATTGCGCCCATTAGTATTCTAGTGGGCATGCCTGTTCGAGCGTCATTTCAACCCTTAAGCCTAGCTTAGTGTTGGGAGCCTACTGCTTTTACTAGCTGTAGCTCCTGAAATACAACGGCG
GATCTGCGATATCCTCTGAGCGTAGTAATTTTTATCTCGCTTTTGACTGGAGTTGCAGCGTCTTTAGCCGCTAAATCCCCCAATTTTTAATGGTTGACCTCGGATCAGGTAGA

16. ***Xylaria* sp. (100%),** Sequence ID PX920275, (s8871-9045) (175 bp)

AAGAGTTCTATAACTCCCAAACCCATGTGAACATACTTAACGTTGCCTCGGCAGGTCACGCCTACCTCGTAGTGCCCTACCCTGTAGGGCCTACCCGGGAGACGTGGTTCAGCCTGCCG
GCGGCCTACCAAACTCTGTTTGATATTGAATTCTGAACCTATAACTAAATAAGTTA

Xylaria flabelliformis, Sequence ID PQ632332.1, 587 bp., Percent identity 100%

AAGTAAAAGTCGTAACAAGGTCTCCGTTGGTGAACCAGCGGAGGGATCATTAAAGAGTTCTATAACTCCCAAACCCATGTGAACATACTTAACGTTGCCTCGGCAGGTCACGCCTACC
TCGTAGTGCCCTACCCTGTAGGGCCTACCCGGGAGACGTGGTTCAGCCTGCCGGCGGCCTACCAAACTCTGTTTGATATTGAATTCTGAACCTATAACTAAATAAGTTAAAACTTTCAA
CAACGGATCTCTTGGTTCTGGCATCGATGAAGAACGCAGCGAAATGCGATAAGTAATGTGAATTGCAGAATTCAGTGAATCATCGAATCTTTGAACGCATATTGCGCCCATTAGTATTC
TAGTGGGCAGGACCGTTCGAGCGTCATTTCGACCCTTAAGCCTTGGTTGCTTAGTGTTGGGAGCCTACGGCAACGTAGCTCCTCAAAGTTAGTGGCGGAGTTGGTTCACACTCTAGAC
GTAGTAGATTTTTATCTCGCCTATCAGTTGGACCGGTCCCCTGCCGTAAAACCCCCCAACTTCTTAAAGTTGACCTCGAATCGGTTCAGACAAACTCGCTAAATTGAAGCATA

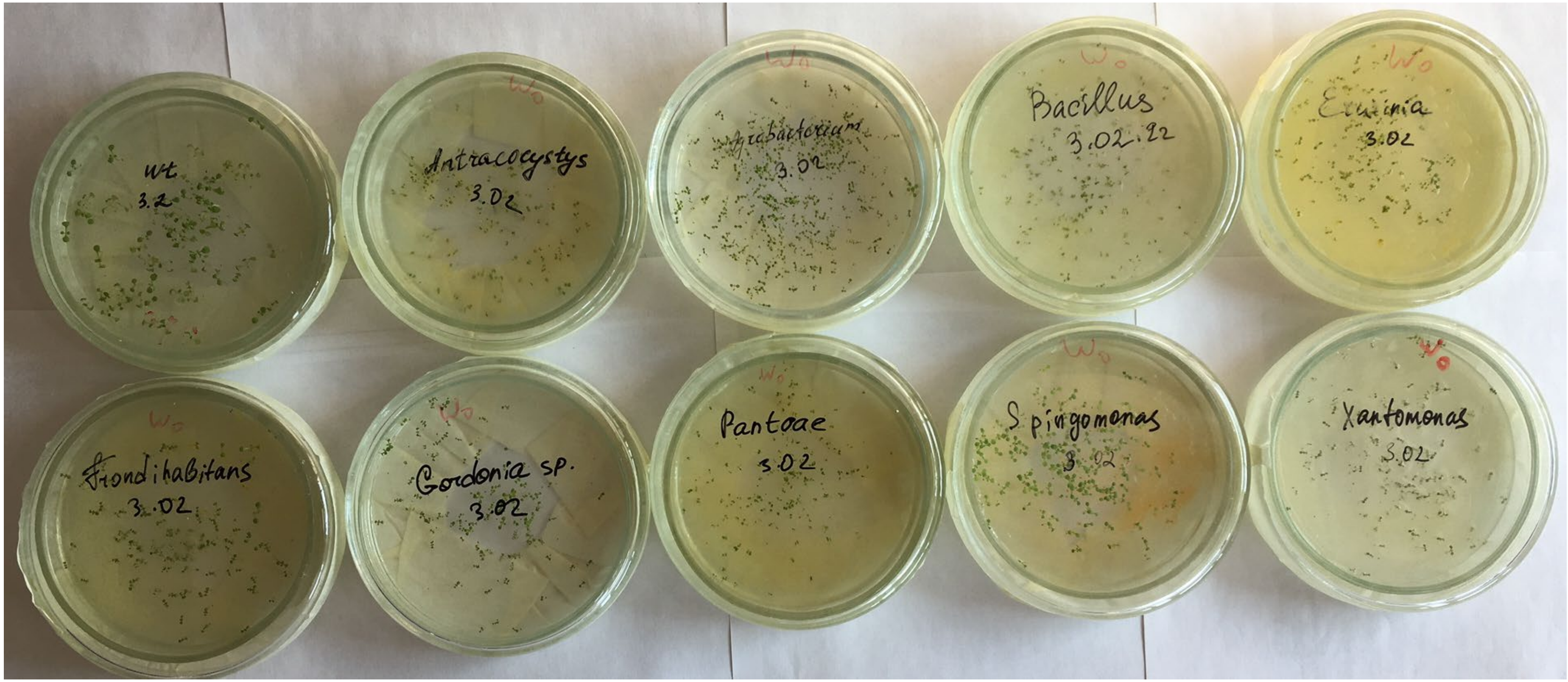

Supplementary Figure S1. *Arabidopsis* seed germination with endophytic grapevine bacteria *Vitis amurensis*

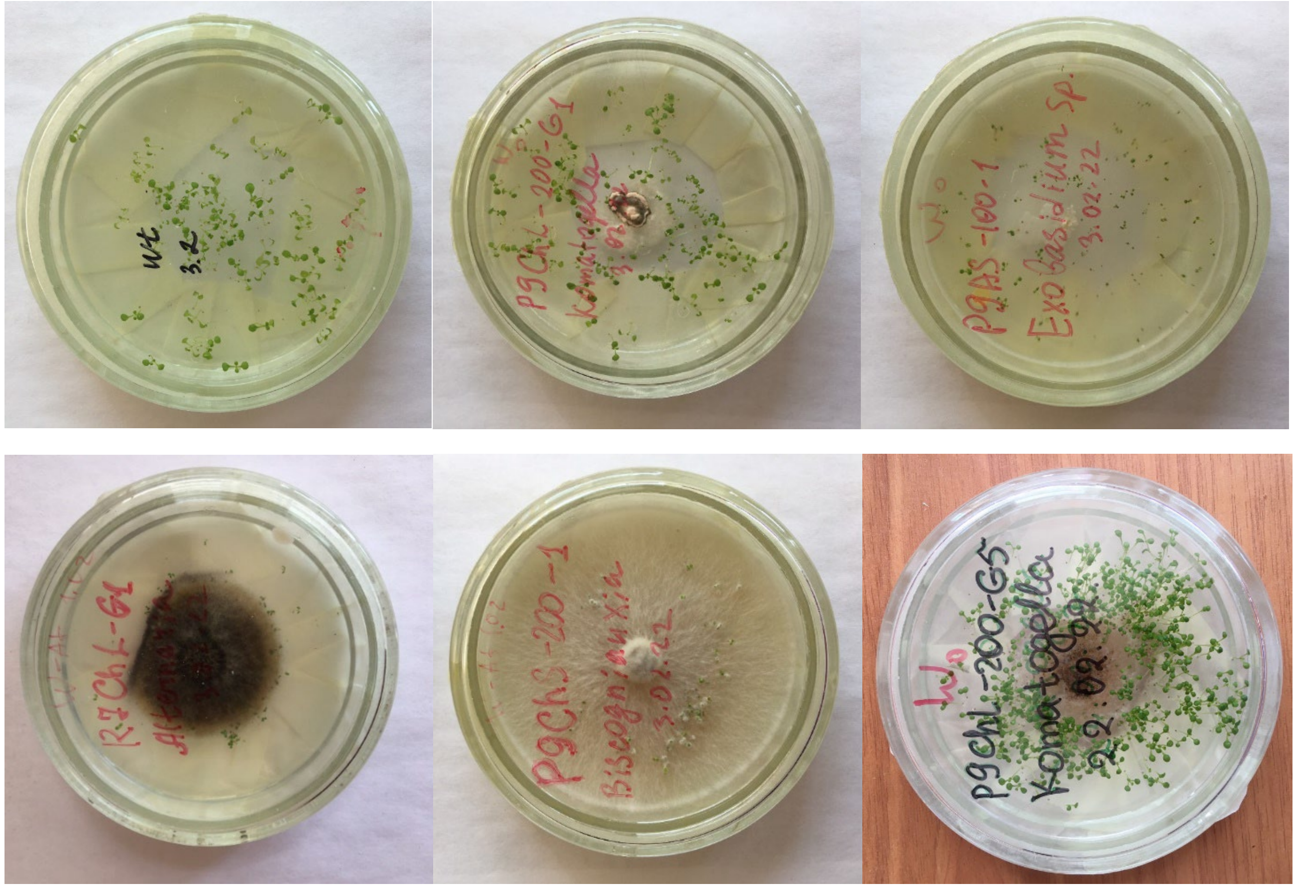

Supplementary Figure S2. *Arabidopsis* seed germination with endophytic grapevine fungi *Vitis amurensis*

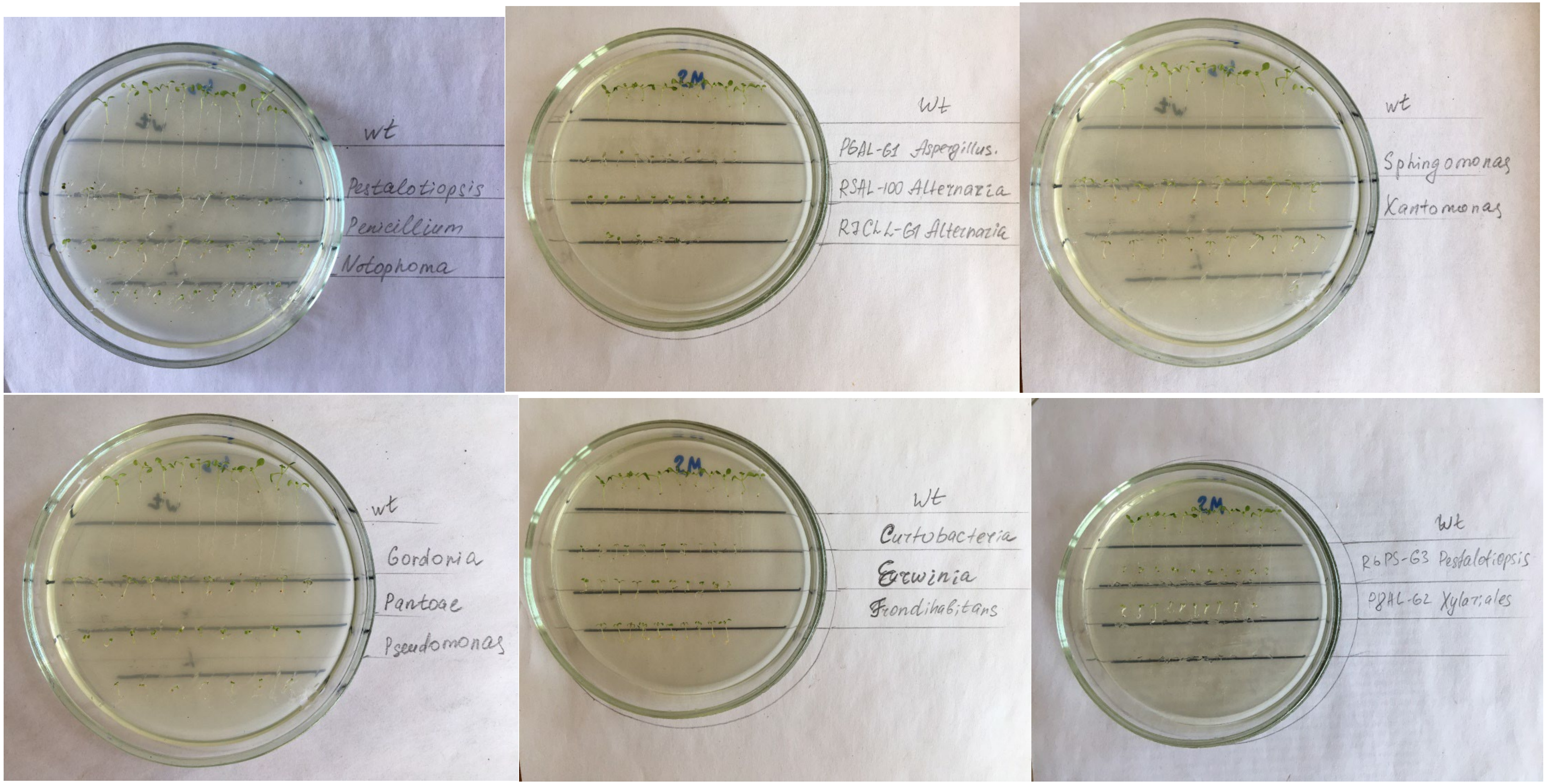

Supplementary Figure S3. Measurement of root length and stem height of *Arabidopsis* seedlings germinated with *Vitis amurensis* grapevine endophytes

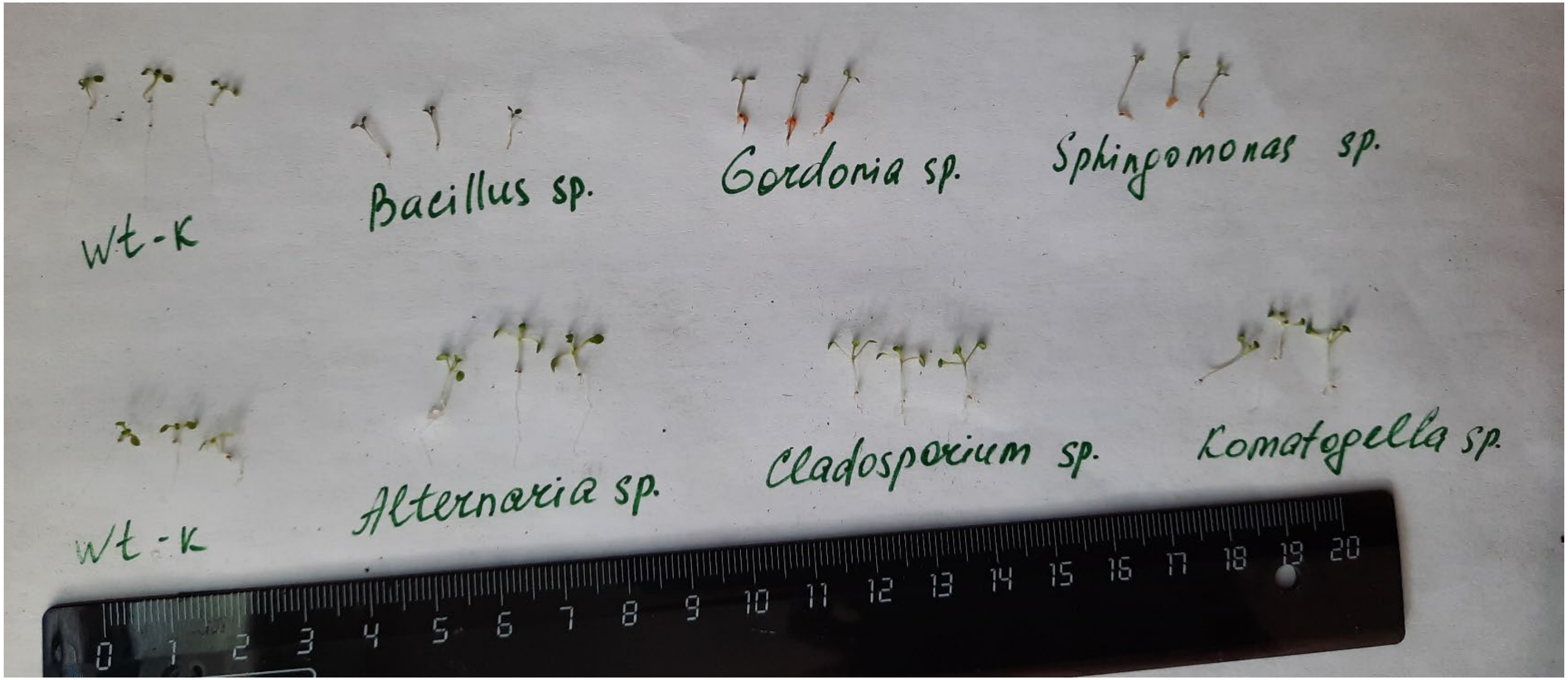

Supplementary Figure S4. Measurement of root length and stem height of *Arabidopsis* seedlings germinated with *Vitis amurensis* grapevine endophytes
